# The association between recreational screen time and cancer risk: findings from the UK Biobank, a large prospective cohort study

**DOI:** 10.1186/s12966-020-00997-6

**Published:** 2020-08-03

**Authors:** Ruth F. Hunter, Jennifer M. Murray, Helen G. Coleman

**Affiliations:** 1 Centre for Public Health, Royal Victoria Hospital, Queen’s University Belfast, Grosvenor Road, Belfast, Northern Ireland BT12 6BJ UK; 2 Centre for Public Health and Patrick G. Johnston Centre for Cancer Research, Queen’s University Belfast, Belfast, Northern Ireland UK

**Keywords:** Sedentary behaviour, Screen time, Cancer, Cohort study, Epidemiology

## Abstract

**Background:**

Evidence is suggestive of sedentary behaviour being associated with an increased risk of endometrial cancer, but the evidence base is too limited to draw any conclusions for other cancers. The aim of the study was to investigate the association between recreational screen time and site-specific cancer risk.

**Methods:**

We analysed data from the prospective UK Biobank cohort study. Cox proportional hazards models were used to estimate hazard ratios (HRs) and 95% confidence intervals (CIs) for associations between daily recreational screen time (including television (TV) viewing time, computer use time and total screen time) and site-specific cancer risk. Partition models and isotemporal substitution models investigated the impact of substituting recreational screen time with physical activity.

**Results:**

During a mean follow-up of 7.6 years, 28,992 incident cancers were identified among 470,578 adults. A 1-h increase in daily TV viewing time was associated with higher risks of oropharyngeal, oesophago-gastric and colon cancer in fully adjusted models. Participants who reported ≤1, compared with 1- ≤ 3, hours/day of TV viewing time had lower risks of lung, breast, and oesophago-gastric cancer. Findings were inconsistent for daily recreational computer use and daily total recreational screen time. The majority of observed associations were small, and were attenuated after excluding cancers diagnosed within the first two years of follow-up, except for oesophago-gastric and colon cancers (HR 1.05, 95% CI: 1.01, 1.10; and HR 1.04, 95% CI: 1.01, 1.07 per 1-h increase in daily TV viewing time, respectively). However, isotemporal substitution models showed reduced risk of some site-specific (oropharyngeal, lung, breast and colorectal) cancers when replacing 1-h/day of TV viewing with 1-h of moderate-intensity physical activity or walking.

**Conclusions:**

Our findings show that daily recreational screen time, particularly TV viewing, was associated with small increased risks of oesophago-gastric and colon cancer. Replacing 1-h/day of TV viewing with 1-h of moderate-intensity physical activity or walking was associated with lower risk of oropharyngeal, lung, breast and colorectal cancers. Further research from other large prospective cohort studies is required, while mechanistic research is warranted to enhance the biological plausibility of these findings.

## Introduction

Research in sedentary behaviours has grown rapidly over recent years [1]. Such behaviours are seen as distinct from physical inactivity or sleep, and have been defined as “any waking behaviour characterised by an energy expenditure ≤1.5 metabolic equivalents (METs), while in a sitting, reclining or lying posture” [1, 2]. This definition is typically operationalised as self-reported sitting (including in recreational and occupational activities), television (TV) viewing or other screen-time. The most recent UK Chief Medical Officers’ Physical Activity Guidelines lists behaviours such as TV viewing and computer-use as examples of sedentary behaviour, highlighting that self-reported screen time is among the most common measures of sedentary behaviour cited in the literature [3]. Screen-time can take many forms including social media use, internet use, gaming, general Smartphone use, watching TV and computer use (regardless of what these devices are used for) [4].

The UK Government guidance on sedentary behaviours, published in 2011 and 2019, suggests that we should minimise time spent in prolonged sedentary behaviours for health benefits [3, 5]. However, owing to the relative early stage of the evidence base, no further recommendations were provided around a timeframe for what would be deemed a ‘harmful’ level of sedentary time exposure. Even the most recent US guidance published in 2018 does not provide more specific recommendations for minimising sedentary time [6].

Evidence demonstrates that prolonged sedentary time is associated with increased risk of non-communicable diseases (NCDs). Mechanistically, sedentary behaviour is thought to impact particularly on cardio-metabolic diseases through adverse effects on lipid and glucose metabolism [7, 8]. Recent evidence from a meta-analysis has demonstrated a significant direct association between 6 and 8 h daily sedentary time and increased all-cause mortality, cardiovascular disease mortality and Type 2 Diabetes Mellitus risk [9]. Prolonged sedentary behaviour is therefore a significant burden on our healthcare systems. In 2016–2017, for example, it was estimated to cost the UK National Health Service £0.8 billion [10].

However, much less is known about sedentary behaviour and cancer, and known biological mechanisms are less well understood [11]. The World Cancer Research Fund/American Institute for Cancer Research (WCRF/AICR) global report in 2018 stated that evidence on sedentary behaviours is limited but is suggestive as being associated with an increased risk of endometrial cancer (pooled risk estimate from three studies comparing the highest versus lowest levels of sitting time was 1.46, 95% CI: 1.21, 1.76, cases = 1579) [11–14]. The evidence base was deemed to be too limited to draw any conclusions for other cancers [11]. However, in a 2018 meta-analysis, Patterson et al., demonstrated significant linear associations of TV viewing with cancer mortality (*N* = 4 studies; relative risk [RR] 1.02, 95% CI: 1.01, 1.03 per 1-h increase in TV viewing/day) [9].

More recent evidence from analyses of the large, prospective UK Biobank cohort shows mixed evidence for an association between sedentary behaviour and cancer outcomes [15]. Celis-Morales et al. (2018) found significant associations of discretionary (or recreational) screen-time (time spent in TV viewing or computer screen use during leisure time) exposure and all-cause mortality (hazard ratio [HR] 1.06, 95% CI: 1.05, 1.07), and cancer incidence (HR 1.04, 95% CI: 1.03, 1.04). This study also found that the associations between longer recreational screen time and adverse health outcomes were strongest for participants with low physical activity, cardiorespiratory fitness and grip strength and markedly attenuated for participants with the highest levels of physical activity, cardiorespiratory fitness and grip strength [15]. Our research group have previously found no evidence for an association between recreational screen time and oesophago-gastric cancer risk within the UK Biobank cohort [16]. In contrast, higher levels of TV viewing time were associated with a greater risk of colon cancer in the same study population (HR for ≥5 h/day vs ≤ 1 h/day = 1.32, 95% CI: 1.04, 1.68) [17], although time spent using computers (excluding using a computer at work) was not associated with colorectal cancer risk in the UK Biobank cohort [17]. The findings of a 2017 meta-analysis including six studies also demonstrated significant associations between the highest compared with the lowest levels of occupational sedentary behaviour, and risk of colon cancer (pooled RRs 1.44, 95% CI: 1.28, 1.62) [18]. On the other hand, there was little evidence of an association between sedentary behaviour and rectal cancer risk [18].

Many of the previous studies investigating the association between sedentary behaviour and health outcomes have attempted to adjust for physical activity levels in their analysis. A recent US Government report has highlighted limited evidence on the role of physical activity in displacing the mortality risks associated with sedentary behaviour [6]. Previous research has demonstrated that high levels of physical activity can attenuate the risks associated with sedentary behaviour [19, 20]. An improved understanding of these interactive effects would enable more specific recommendations to be made regarding quantifying prolonged sedentary time. Much of the previous research has modelled the joint effects of physical activity and sedentary behaviour [20]. However, other analytical approaches such as isotemporal substitution and partition models [19], enable us to model replacing sedentary behaviour with physical activity which may be a more time efficient method of promoting healthy behaviour. Analytical techniques such as partition models and isotemporal substitution models [21] could help to model such predictions, but have yet to be extensively applied in large cohort analyses.

Therefore, this study aimed to add to the relatively scant evidence base [11] by investigating daily recreational screen time (including TV viewing, computer use and total screen-use) in relation to the risk of site-specific cancers in the large UK Biobank cohort study. Partition and isotemporal substitution models were also used to investigate the impact of substituting recreational screen time with physical activity, in relation to site-specific cancer risk.

## Methods

### Study design

Between 2006 and 2010, UK Biobank recruited a cohort of 502,619 adults (5.5% response rate) aged 40–69 years from the general population [22, 23]. Approximately 9.2 million invitations were mailed to potential participants who were registered with the National Health Service (NHS) and living within a 25-mile radius of one of the 22 assessment centres across England, Scotland and Wales.

From this overall cohort, we excluded participants if: [1] they had been diagnosed with malignant cancer (excluding non-melanoma skin cancer) at baseline (*n* = 26,868); and [2] they did not complete the self-report assessments of their daily TV viewing time (*n* = 5078), daily recreational computer time (*n* = 8000) or daily total recreational screen time (*n* = 11,232) [3]; they requested to be removed from the UK Biobank dataset as per General Data Protection Regulation (GDPR) (*n* = 95). This resulted in 470,578 participants being included in the analysis for daily TV viewing time, 467,656 participants being included in the analysis for daily recreational computer time and 464,424 participants being included in the analysis for daily total recreational screen time. All participants provided informed consent.

### Screen time assessment

We have used daily TV viewing time as our primary exposure. Firstly, TV viewing time was almost three times more prevalent as a recreational sedentary behaviour than computer use within this UK population. Secondly, we were concerned that using daily total recreational screen time as the primary analysis may overestimate total screen time through double counting (if participants watched TV and used computers at the same time). Therefore, we did not feel that total screen time was an appropriate focus for our primary analysis. Thirdly, as acknowledged in a recent study [24], a large body of research has focussed on TV viewing as a primary exposure representing a *type of* sedentary behaviour, demonstrating consistent associations, particularly with CVD risk, in population cohort studies akin to the UK Biobank. Patterson et al., (2018) also highlights that daily TV viewing time may show stronger associations with health outcomes, and also may be one of the most amenable types of sedentary behaviour [9].

Relevant screen-time exposure variables were assessed by self-reported time spent watching TV, and time spent using the computer outside of work, which were used to derive daily total recreational screen time. Self-reported TV viewing time was assessed for all participants by asking the following question: “In a typical DAY, how many hours do you spend watching TV? (Put 0 if you do not spend any time doing it)?” Self-reported daily recreational computer use time was assessed for all participants by asking the following question: “In a typical DAY, how many hours do you spend using the computer? (Do not include using a computer at work; put 0 if you do not spend any time doing it).” Durations of < 0 h were set to missing, as were responses of “Do not know” or “Prefer not to answer”. If the respondent replied “Less than an hour a day”, this was recoded to 0.5 h. Daily total recreational screen time was then computed as the sum of hours spent watching TV and hours spent using the computer. If the summation of total hours spent watching TV and hours spent using the computer was greater than 24, this was set to missing (*n* = 35).

### Physical activity assessment

Self-report physical activity was assessed for all participants using the validated short-form International Physical Activity Questionnaire (IPAQ) [25] on which participants reported the frequency (i.e. days/week) and duration (i.e. minutes/day) of walking, moderate- and vigorous-intensity physical activity in the past seven days. For each domain (walking, moderate, vigorous), durations of < 10 min/day were recoded to 0 and durations of > 180 min were truncated at 180 min/day in line with IPAQ processing rules. This was used to derive hours/day spent in walking, moderate- and vigorous-intensity physical activity. A categorical variable was derived representing participants’ IPAQ physical activity category and participants were classified as ‘low’ (not meeting criteria for the ‘moderate’ or ‘high’ categories), ‘moderate’ (at least 20 min of vigorous-intensity physical activity on three or more days/week; at least 30 min of moderate-intensity physical activity or walking on five or more days/week; five or more days/week spent in any combination of walking, moderate- or vigorous-intensity physical activity achieving at least 600 MET minutes/week) or ‘high’ (at least three days/week of vigorous-intensity physical activity achieving at least 1500 MET minutes/week; at least seven days/week spent in any combination of walking, moderate- or vigorous-intensity physical activity achieving at least 3000 MET minutes/week) activity levels. All data processing was carried out according to official IPAQ rules [26].

### Assessment of covariates

Height (m), weight (kg), and waist and hip circumference (cm) were measured by staff at the UK Biobank study centre. Body mass index (BMI) was then calculated from the weight and height measurements (kg/m^2^). Waist circumference measurements were taken from the level of the umbilicus and regarded as a measure of central obesity, using official cut-off values established by the International Diabetes Federation (> 94 cm in men and > 80 cm in women) [27]. Age, sex and postcodes were acquired from a central registry for all participants and updated by the participant. Participants also self-reported their ethnicity, educational attainment, lifestyle behaviours (smoking status, alcohol consumption, dietary intake, and sunscreen/ultraviolet (UV) protection use) and medical history using electronic questionnaires. Townsend deprivation scores were derived from postcodes [28]. Core confounders for all models included socio-demographic factors (i.e. age, sex, ethnicity, educational attainment and deprivation index), smoking status, alcohol consumption, fruit and vegetable consumption, BMI, height and waist-hip ratio. Cancer site-specific confounders included use of sun/UV protection (melanoma), self-reported oesophageal reflux (oesophagus cancer), diabetes at baseline (pancreatic and colorectal cancers), aspirin use (colorectal cancers), red and processed meat intake (colorectal cancers), hormone replacement therapy (HRT) use (breast, uterus and colorectal cancers), oral contraceptive use (breast and uterus cancers), number of live births (breast and uterus cancers), age at menarche (breast and uterus cancers), age at menopause (breast and uterus cancers), hysterectomy status (breast and uterus cancers) and self-reported family history of cancer (lung, prostate, and breast cancers), based on known aetiological risk factors for these tumours.

Proportions of missing data were less than 1% for all variables apart from aspirin use (1.9%), red meat intake (1.1%), age at menarche (1.6%), age at menopause (2.1%), education (1.5%), fruit and vegetable consumption (2.6%), hysterectomy status (5.9%), family history of cancer (1.5%), daily moderate-intensity physical activity (14.0%), daily vigorous-intensity physical activity (10.5%) and daily walking time (12.7%). In multivariable models adjusting for the specific factors listed, we conducted a complete-case analysis restricted to individuals who did not have missing information.

### Cancer ascertainment

For the present analysis, the main outcomes were incident site-specific cancers. Incident cancers for participants in the UK Biobank cohort were identified through records maintained at national cancer registries (Health and Social Care Information Centre and the NHS Central Register) and identified from the International Classification of Diseases, 9th and 10th revisions (ICD-9 and ICD-10 [29]). Cancer outcomes were coded according to ICD-9 and ICD-10 as follows: melanoma (ICD-10: C43; ICD-9: 172), oropharyngeal cancers (ICD-10: C00-C14; ICD-9: 140–149), lung (ICD-10: C33-C34; ICD-9: 162), breast: female only (ICD-10: C50; ICD-9: 174), uterus (ICD-10: C54; ICD-9: 182), ovary (ICD-10: C56; ICD-9: 183), prostate (ICD-10: C61; ICD-9: 185), oesophagus (ICD-10: C15; ICD-9: 150), stomach (ICD-10: C16; ICD-9: 151), hepatobiliary tract (ICD-10: C22-C24; ICD-9: 155–156), pancreatic (ICD-10: C25; ICD-9: 157), kidney (ICD-10: C64-C65; ICD-9: 189.0–189.1), bladder (ICD-10: C66-C67; ICD-9: 188, 189.2), colorectal (ICD-10: C18-C21; ICD-9: 153–154), colon (ICD-10: C18; ICD-9: 153), rectum (ICD-10: C19-C20; ICD-9: 1540–1541), brain tumours (ICD-10: C71; ICD-9: 191), thyroid (ICD-10: C73; ICD-9: 193), and haematological malignancies (ICD-10: C81-C96; ICD-9: 200–208), including separate analysis of non-Hodgkin’s lymphoma (ICD-10: C82-C85; ICD-9: 200, 202).

### Statistical analyses

Our statistical analyses addressed the following research questions:

1. What is the association between daily recreational screen time (i.e. TV viewing, computer use and total screen time) and site-specific cancers (including endometrial, colorectal, pre- and post-menopausal breast, prostate, lung, and other cancers)?

2. How do these associations vary by gender, age, socio-economic status, smoking and excess body weight?

3. What is the effect of replacing TV viewing time with physical activity on site-specific cancer risk?

Descriptive statistics for all covariates are presented according to participants’ total daily TV viewing time. Categorical variables are presented as participant numbers and percentages. Means and standard deviations (SDs) are presented for continuous variables. Follow-up time in days from baseline was used as the timescale, and for each participant end of follow-up occurred at: [1] cancer diagnosis date [2]; date of emigration; (3) date of death; or (4) end of follow-up (14th December 2016), whichever came first.

Cox proportional hazards models were used to estimate hazard ratios (HRs) and 95% confidence intervals (CIs) showing the relationship between a 1-h increase/day in TV viewing time and cancer. All analyses were adjusted for age and sex in the baseline model. Additional covariates were added in the second adjusted model and included ethnicity (white/other), deprivation index (quintiles), education (University degree, A-levels/HNC/HND/NVQ, GCSE/O-level/CSE, Other, None), BMI (kg/m^2^), height (m), smoking status (never, former light smoker [< 20 pack-years], former heavy smoker [≥20 pack-years], current light smoker [< 20 pack-years], current heavy smoker [≥20 pack-years]), alcohol intake (never, former, current [<once/week], current [≥once/week]) and fruit and vegetable intake (< 5 portions/day, ≥5 portions/day). Cancer site-specific covariates were included in the third adjusted model for each type of cancer (details included in the footnotes of Tables 2-6). These included use of sun/UV protection, HRT use, oral contraceptive use, number of live births, age at menarche, age at menopause, hysterectomy status, diabetes at baseline, aspirin use, red meat intake, and processed meat intake. For analyses including gender-specific covariates (e.g. colorectal cancer, colon cancer and rectum cancer), separate models were run for males and females and HRs were combined using inverse variance meta-analysis and a fixed-effects model [30–32]. Participants were excluded from the analysis if they did not have the complete exposure and covariate data required for each model. We did not adjust for total dietary energy intake as the large amount of missing data (for 57.6% of participants) made this unfeasible. Further analyses were conducted to investigate the role of central adiposity by running all models with and without adjustment for waist-hip ratio. Models including incident breast cancer, prostate cancer and lung cancer were run with and without adjustment for self-report family history (mother, father, siblings). The oesophageal cancer model was also run with and without adjustment for self-reported gastro-oesophageal reflux disease (GORD).

These analyses were repeated separately to investigate the relationship between a 1-h increase/day in [1] daily recreational computer time; (2) daily total recreational screen time; and site-specific cancer risk. To characterise the dose-response relationships [9, 33], we repeated these analyses with categorised independent variables as follows: daily TV viewing time (1- ≤ 3 h [reference category]; ≤1 h; 3- ≤ 5 h; > 5 h), daily recreational computer use time (≤1 h [reference category]; none; 1- ≤ 3 h; > 3 h) and daily total recreational screen time (1- ≤ 4 h [reference category]; ≤1 h; 4- ≤ 8 h; > 8 h) categorised based on previously published categories [17].

A series of partition models and isotemporal substitution models [21] were used for each type of cancer to examine the associations of daily TV viewing time, time spent walking/day, time spent in moderate-intensity physical activity/day, time spent in vigorous-intensity physical activity/day and cancer incidence [21, 34–37]. Partition models examined all behaviours simultaneously, without adjusting for total physical activity time. Therefore, the HR for one type of physical activity represented the effect of increasing this type of physical activity (by 1-h/day) while holding the other physical activities constant. Since total physical activity time is not included in the model (and thus is not held constant), these results represent the effect of adding a behaviour (i.e. walking, moderate-activity, vigorous-activity, TV screen time) whilst holding the others constant. The effects of substituting one behaviour type by another for the same amount of time (i.e. replacing 1-h/day of TV screen time for 1-h/day of walking, moderate-intensity physical activity or vigorous-intensity physical activity) was investigated using isotemporal substitution models which adjusted for time spent walking/day, time spent in moderate-intensity physical activity/day, time spent in vigorous-intensity physical activity/day and total activity time/day (i.e. the summation of walking, moderate activity, vigorous activity and TV viewing time). In this case, since total activity is included the model (and thus is held constant), these results represent the effect of replacing daily TV viewing time with the same amount of another physical activity type (i.e. walking, moderate- or vigorous- activity) while holding the others constant.

Sensitivity analyses were conducted by confining the analysis to cancers diagnosed at least two years following baseline to examine the impact of removing prevalent disease. Subgroup analyses were conducted by selected baseline characteristics (supplement 1/Table 1.2–1.9). These included sex, age, deprivation index, smoking status, BMI (with reference to obese/non-obese thresholds defined for various ethnic groups by gender in a previous UK Biobank study [38], assuming that participants with mixed backgrounds or ‘other’ ethnicities had the same obesity thresholds as white participants since cut-off points were not available for this group), and IPAQ physical activity category. Further analyses were conducted by creating four categories based on body fat percentage and physical activity levels defined according to the IPAQ (High/moderate physical activity and low/optimal body fat percentage; Low physical activity and low/optimal body fat percentage; High/moderate physical activity and high body fat percentage; Low physical activity and high body fat percentage), in order to investigate any differential associations between sedentary behaviour and cancer risk according to the ‘fat but fit’ hypothesis [39]. Body fat percentage cut-points were derived from previously established thresholds defined by age, gender, ethnicity and BMI [40]. We assumed participants with mixed backgrounds or ‘other’ ethnicities had the same body fat percentage thresholds as white participants. Subgroup analyses were also conducted by menopausal status for female-specific cancers (i.e. breast, uterus, ovary cancers). Interactions were tested using the Wald test for homogeneity and declared significant if *p* < 0.01 in line with previous studies [41].

**Table 1:** Baseline characteristics by self-report daily TV viewing time. Values are numbers and percentages unless otherwise stated

			Total TV viewing time							
	Total		≤1 h per day		1- ≤ 3 h per day		3- ≤ 5 h per day		> 5 h/day	
	No./mean	%/SD	No./mean	%/SD	No./mean	%/SD	No./mean	%/SD	No./mean	%/SD
**Total participants**	470,578	100.0%	97,419	20.7%	236,988	50.4%	110,334	23.5%	25,837	5.5%
**Self-report total screen time (hours/day; mean/SD)**	3.9	2.1	2.0	1.5	3.5	1.4	5.3	1.4	7.9	2.4
Time spent watching TV (hours/day; mean/SD)	2.8	1.7	0.7	0.4	2.5	0.5	4.3	0.5	6.9	1.8
Time spent using computers (hours/day; mean/SD)	1.1	1.4	1.2	1.5	1.1	1.3	1.0	1.3	1.1	1.7
**IPAQ physical activity (mean/SD)**										
Hours/day of walking	0.75	0.78	0.71	0.74	0.76	0.79	0.77	0.79	0.67	0.74
Hours/day of moderate-intensity physical activity	0.56	0.72	0.54	0.70	0.57	0.73	0.58	0.74	0.46	0.67
Hours/day of vigorous-intensity physical activity	0.20	0.35	0.22	0.34	0.20	0.35	0.18	0.35	0.14	0.33
**Age at baseline (mean/SD)**	56.3	8.1	54.2	8.0	55.9	8.1	58.5	7.6	59.0	7.6
**Height (m) (mean/SD)**	1.7	0.1	1.7	0.1	1.7	0.1	1.7	0.1	1.7	0.1
**Sex**										
Female	253,188	53.8%	53,500	54.9%	127,135	53.7%	59,553	54.0%	13,000	50.3%
Male	217,390	46.2%	43,919	45.1%	109,853	46.4%	50,781	46.0%	12,837	49.7%
**Ethnicity**										
White	443,484	94.6%	90,405	93.3%	224,142	94.9%	104,922	95.4%	24,015	93.3%
Black	7505	1.6%	1549	1.6%	3358	1.4%	1846	1.7%	752	2.9%
South Asian	9395	2.0%	2582	2.7%	4721	2.0%	1639	1.5%	453	1.8%
Chinese	1501	0.3%	465	0.5%	717	0.3%	266	0.2%	53	0.2%
Mixed background or others	7069	1.5%	1949	2.0%	3299	1.4%	1354	1.2%	467	1.8%
**Townsend deprivation quintile**										
1 (Least deprived)	94,590	20.1%	19,860	20.4%	51,164	21.6%	20,497	18.6%	3069	11.9%
2	93,950	20.0%	18,854	19.4%	49,804	21.0%	21,691	19.7%	3601	14.0%
3	94,166	20.0%	18,857	19.4%	48,706	20.6%	22,379	20.3%	4224	16.4%
4	94,118	20.0%	20,209	20.8%	46,505	19.7%	22,081	20.0%	5323	20.6%
5 (Most deprived)	93,165	19.8%	19,530	20.1%	40,495	17.1%	23,561	21.4%	9579	37.1%
**Education**										
University degree	153,223	33.1%	52,250	54.3%	79,041	33.9%	19,257	17.8%	2675	10.6%
A-levels/HNC/HND/NVQ	83,315	18.0%	15,934	16.6%	44,649	19.1%	18,921	17.5%	3811	15.1%
GCSE/O-level/CSE	124,765	26.9%	17,518	18.2%	66,180	28.3%	34,257	31.6%	6810	27.0%
Other	24,018	5.2%	4230	4.4%	12,530	5.4%	6091	5.6%	1167	4.6%
None	78,028	16.8%	6346	6.6%	31,125	13.3%	29,800	27.5%	10,757	42.7%
**Smoking status**^**a**^										
Never	257,696	55.0%	58,981	60.7%	132,976	56.3%	54,858	49.9%	10,881	42.4%
Former light smoker	119,085	25.4%	24,556	25.3%	61,147	25.9%	27,891	25.4%	5491	21.4%
Former heavy smoker	42,251	9.0%	5350	5.5%	19,256	8.2%	13,521	12.3%	4124	16.1%
Current light smoker	27,794	5.9%	5535	5.7%	13,706	5.8%	6646	6.1%	1907	7.4%
Current heavy smoker	22,082	4.7%	2735	2.8%	9094	3.9%	6972	6.3%	3281	12.8%
**Alcohol intake**										
Never	20,749	4.4%	4873	5.0%	9428	4.0%	4868	4.4%	1580	6.1%
Former drinker	16,659	3.5%	3330	3.4%	7128	3.0%	4342	3.9%	1859	7.2%
Current drinker: <once/week	106,020	22.6%	19,466	20.0%	50,958	21.5%	27,939	25.3%	7657	29.7%
Current drinker: ≥once/week	326,759	69.5%	69,676	71.6%	169,324	71.5%	73,089	66.3%	14,670	56.9%
**Dietary intake (mean/SD)**										
Fruits and vegetables (portion/day)	4.7	3.1	5.1	3.2	4.7	3.0	4.5	3.0	4.2	3.3
Red meat (portion/week)	2.1	1.5	2.0	1.4	2.1	1.4	2.2	1.5	2.4	1.7
Processed meat (portion/week)	1.5	1.4	1.3	1.4	1.5	1.4	1.6	1.4	1.9	1.6
**Body Mass Index (Kg/m**^**2**^**) (mean/SD)**	27.4	4.8	26.0	4.3	27.3	4.6	28.5	4.9	29.7	5.8
**Body Mass Index (Kg/m**^**2**^**)**										
< 18.5	2418	0.5%	825	0.9%	1113	0.5%	352	0.3%	128	0.5%
18.5- < 25	152,533	32.6%	44,075	45.5%	77,507	32.9%	26,157	23.8%	4794	18.8%
25- < 30	199,212	42.6%	37,528	38.7%	103,141	43.7%	48,586	44.3%	9957	39.1%
30+	113,922	24.3%	14,489	15.0%	54,197	23.0%	34,626	31.6%	10,610	41.6%
**Body fat percentage (mean/SD)**	31.3	8.5	28.9	8.3	31.1	8.4	33.2	8.4	34.3	8.8
**Waist:hip ratio**^**b**^										
Waist:hip ratio (mean/SD)	0.9	0.1	0.9	0.1	0.9	0.1	0.9	0.1	0.9	0.1
Below IDF guideline	202,545	43.2%	54,750	56.4%	104,482	44.2%	36,717	33.4%	6596	25.7%
Above IDF guideline	266,443	56.8%	42,333	43.6%	131,829	55.8%	73,249	66.6%	19,032	74.3%
**Health status**										
Diabetes^c^	24,347	5.2%	3085	3.2%	10,404	4.4%	7687	7.0%	3171	12.4%
Gastro-oesophageal reflux^d^	22,495	4.8%	3233	3.3%	10,672	4.5%	6648	6.0%	1942	7.5%
**Family history**^**e**^										
Prostate cancer	37,225	8.0%	8431	8.8%	18,607	8.0%	8332	7.7%	1855	7.3%
Breast cancer	49,524	10.7%	10,520	10.9%	24,986	10.7%	11,360	10.5%	2658	10.5%
Lung cancer	59,042	12.7%	9596	10.0%	29,218	12.5%	16,107	14.9%	4121	16.3%
Bowel cancer	52,109	11.2%	10,181	10.6%	25,851	11.1%	12,943	11.9%	3134	12.4%
**Use of sun/UV protection**										
Never/rarely/sometimes	203,968	43.7%	43,450	44.9%	99,121	42.1%	48,286	44.1%	13,111	51.4%
Most of the time/always	260,241	55.7%	52,699	54.5%	135,033	57.4%	60,493	55.3%	12,016	47.1%
Do not go out in sunshine	2770	0.6%	538	0.6%	1136	0.5%	717	0.7%	379	1.5%
**Aspirin use**										
Regularly uses aspirin^f^	64,822	14.0%	9711	10.1%	29,908	12.9%	19,246	17.8%	5957	23.7%
**HRT use**^**g**^										
Ever used HRT	95,369	37.8%	14,791	27.8%	46,587	36.8%	27,639	46.6%	6352	49.1%
**Oral contraceptive use**^**g**^										
Ever taken oral contraceptive pill	205,528	81.4%	44,285	83.0%	104,772	82.7%	46,695	78.7%	9776	75.6%
**Number of live births (0, 1, 2, 3+ live births)**^**g**^**(mean/SD)**	1.8	1.2	1.8	1.2	1.8	1.2	1.9	1.2	2.0	1.3
**Age at menarche (mean/SD)**^**g**^	13.0	1.6	13.0	1.6	13.0	1.6	13.0	1.7	13.0	1.7
**Age at menopause (mean/SD)**^**g**^	49.8	5.1	50.0	4.7	49.9	5.0	49.6	5.4	49.0	5.8
**Menopausal status**^**g**^										
Had menopause	151,101	59.8%	27,736	51.9%	74,075	58.4%	40,399	68.0%	8891	68.6%
Not had menopause	62,570	24.8%	18,659	34.9%	33,174	26.1%	9075	15.3%	1662	12.8%
Unsure	39,065	15.5%	7002	13.1%	19,684	15.5%	9969	16.8%	2410	18.6%
**Hysterectomy status**^**g**^										
Had hysterectomy	17,530	7.8%	2458	5.0%	8193	7.2%	5483	10.6%	1396	12.7%
Not had hysterectomy/unsure	207,953	92.2%	46,846	95.0%	105,238	92.8%	46,232	89.4%	9637	87.4%

CSE: Certificate of Secondary Education; GCSE: General Certificate of Secondary Education; HNC: Higher National Certificate; HND: Higher National Diploma; HRT: hormone-replacement therapy; IDF: International Diabetes Federation; MVPA: moderate-vigorous intensity physical activity; NVQ: National Vocational Qualifications; UV: ultraviolet

^a^Defined in terms of pack-years: light (< 20 pack-years), heavy (≥20 pack-years)

^b^Based on IDF criteria (waist circumference > 94 cm in men; > 80 cm in women)

^c^Diagnosed by doctor

^d^Self-reported

^e^Based on self-reported illnesses of father, mother and siblings

^f^Regular use defined as most days of the week for the last 4 weeks

^g^Female participants only

The proportional hazards assumption was tested for each model formally using Schoenfeld residuals (*p* < 0.05 indicated potential violation of the proportional hazards assumption), and by visual inspection of scaled Schoenfeld residual plots [42] and log-log plots (parallel curves indicated that there was no evidence for violation of the proportional hazards assumption). We used restricted cubic splines analyses to examine the associations between the continuous exposure variables (daily TV viewing time, daily recreational computer time, daily total recreational screen time, daily moderate-intensity physical activity, daily vigorous-intensity physical activity, and daily walking time) and cancer risk at each site for potential violations of linearity assumptions. No serious violation of the linearity assumption was observed. Details are reported in supplement 2. Analyses were carried out using Stata 13 [43].

## Results

Participant characteristics according to total daily TV viewing time are shown in Table 1. Among the 470,578 participants included in this analysis, 53.8% were women and the mean age was 56.3 years. Most participants reported that they spent between 2 and 8 h/day watching TV or using the computer. During a mean follow-up time of 7.6 (SD 1.4) years (median 7.8 years, interquartile range 7.0–8.5), 28,992 incident cancers were identified.

### Association of site-specific cancer risk and daily TV viewing time

**Fig. 1:**
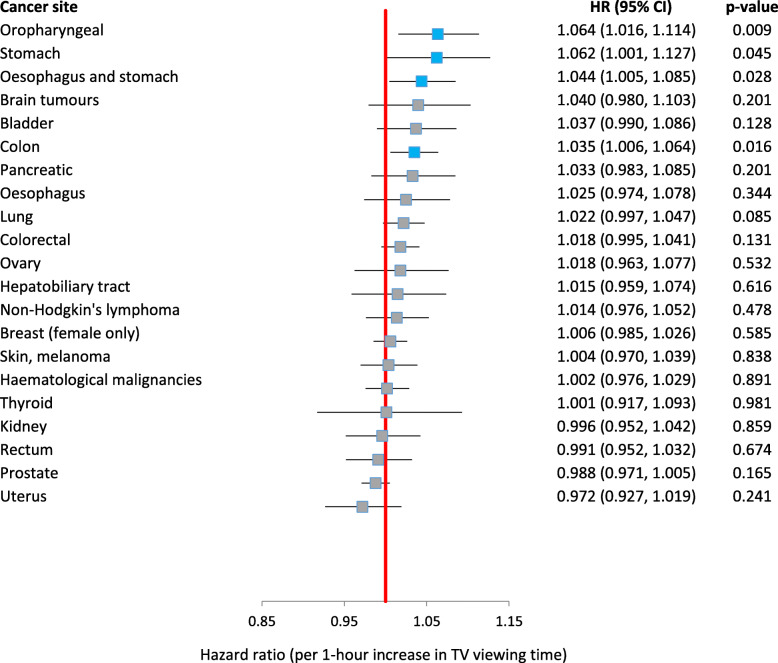
Summary hazard ratio estimates by cancer site

Table 2 and Fig. 1 show the association between daily TV viewing time and site-specific cancer risk. A 1-h increase in daily TV viewing time was associated with higher risk of oropharyngeal cancer (HR 1.06, 95% CI: 1.02, 1.11), stomach cancer (HR 1.06, 95% CI: 1.001, 1.13), oesophagus and stomach cancer (HR 1.04, 95% CI: 1.005, 1.09), and colon cancer (HR 1.04, 95% CI: 1.01, 1.06) in fully adjusted models. In addition, the categorical analysis showed that participants who reported > 5 h/day of TV viewing time had a higher risk of oropharyngeal cancer (HR 1.48, HR: 1.09, 2.01) and a lower risk of uterus cancer (HR 0.61, 95% CI: 0.42, 0.88) compared to participants who reported 1- ≤ 3 h/day of TV viewing time. Participants who reported 3- ≤ 5 h/day of TV viewing time had a higher risk of bladder cancer (HR 1.21, 95% CI: 1.002, 1.45) compared to participants who reported 1- ≤ 3 h/day of TV viewing time, but no dose-response association was evident for greater duration of TV viewing time.

**Table 2:** Results of Cox proportional hazards analyses investigating the association between self-report daily TV viewing time and cancer incidence

		1 h increase in TV viewing time	*p*-value	≤1 h	1- ≤ 3 h (reference)	3- ≤ 5 h	> 5 h
Person-years		3,526,324		736,537	1,781,542	818,674	189,571
Skin, melanoma	Cases	1635		315	831	404	85
	HR (95% CI)*	0.98 (0.95 1.01)	0.24**	0.99 (0.87 1.12)	1.00	0.96 (0.85 1.08)	0.84 (0.67 1.06)
	HR (95% CI)†	1.01 (0.97 1.04)	0.74	0.99 (0.87 1.13)	1.00	1.004 (0.89 1.14)	1.01 (0.80 1.29)
	HR (95% CI)^a^	1.004 (0.97 1.04)	0.84	1.01 (0.88 1.15)	1.00	1.001 (0.88 1.13)	1.02 (0.81 1.30)
Oropharyngeal	Cases	557		86	263	148	60
	HR (95% CI)*	**1.12 (1.08 1.17)**	**< 0.001**	0.83 (0.65 1.06)	1.00	1.17 (0.96 1.43)	**1.99 (1.50 2.63)**
	HR (95% CI)†	**1.06 (1.02 1.11)**	**0.009**	0.83 (0.64 1.07)	1.00	1.07 (0.87 1.32)	**1.48 (1.09 2.01)**
	HR (95% CI)	**1.06 (1.02 1.11)**	**0.009**	0.83 (0.64 1.07)	1.00	1.07 (0.87 1.32)	**1.48 (1.09 2.01)**
Lung	Cases	2076		236	901	656	283
	HR (95% CI)*	**1.17 (1.14 1.19)**	< 0.001**	**0.74 (0.64 0.86)**	**1.00**	**1.29 (1.17 1.43)**	**2.28 (1.99 2.61)**
	HR (95% CI)†	1.02 (0.995 1.04)	0.12**	0.87 (0.75 1.01)	1.00	0.98 (0.88 1.09)	1.09 (0.93 1.26)
	HR (95% CI)^h^	1.02 (0.997 1.05)	0.09**	**0.85 (0.73 0.997)**	1.00	0.98 (0.88 1.09)	1.09 (0.94 1.27)
Breast (female only)	Cases	5702		1097	2903	1386	316
	HR (95% CI)*	1.01 (0.99 1.02)	0.43	0.93 (0.87 1.002)	1.00	0.97 (0.91 1.03)	1.003 (0.89 1.13)
	HR (95% CI)†	1.003 (0.98 1.02)	0.77**	0.94 (0.88 1.01)	1.00	0.96 (0.90 1.03)	0.99 (0.87 1.12)
	HR (95% CI)^b, h^	1.01 (0.99 1.03)	0.59**	**0.92 (0.85 0.996)**	1.00	0.95 (0.88 1.02)	1.01 (0.87 1.16)
Uterus	Cases	872		151	411	264	46
	HR (95% CI)*	1.04 (0.999 1.08)	0.053	0.97 (0.81 1.17)	1.00	**1.21 (1.03 1.41)**	0.95 (0.70 1.28)
	HR (95% CI)†	0.97 (0.93 1.02)	0.21	1.05 (0.86 1.27)	1.00	1.05 (0.89 1.24)	**0.63 (0.44 0.88)**
	HR (95% CI)^c^	0.97 (0.93 1.02)	0.24	1.03 (0.84 1.27)	1.00	1.04 (0.87 1.24)	**0.61 (0.42 0.88)**
Ovary	Cases	578		105	287	155	31
	HR (95% CI)*	1.002 (0.95 1.05)	0.93	0.95 (0.76 1.19)	1.00	1.02 (0.84 1.24)	0.91 (0.63 1.32)
	HR (95% CI)†	1.02 (0.96 1.08)	0.53	0.90 (0.71 1.15)	1.00	1.04 (0.84 1.27)	0.93 (0.63 1.38)
	HR (95% CI)	1.02 (0.96 1.08)	0.53	0.90 (0.71 1.15)	1.00	1.04 (0.84 1.27)	0.93 (0.63 1.38)
Prostate	Cases	5979		1116	2957	1562	344
	HR (95% CI)*	**0.96 (0.95 0.98)**	< 0.001**	**1.08 (1.01 1.15)**	1.00	0.96 (0.90 1.02)	**0.81 (0.73 0.91)**
	HR (95% CI)†	0.99 (0.97 1.004)	0.12**	1.05 (0.98 1.13)	1.00	1.01 (0.94 1.07)	0.94 (0.83 1.06)
	HR (95% CI)^h^	0.99 (0.97 1.01)	0.17**	1.04 (0.97 1.12)	1.00	1.01 (0.95 1.08)	0.95 (0.84 1.07)
Oesophagus	Cases	541		70	246	176	49
	HR (95% CI)*	**1.10 (1.05 1.15)**	**< 0.001**	0.80 (0.61 1.04)	1.00	1.30 (1.07 1.58)	1.44 (1.06 1.96)
	HR (95% CI)†	1.03 (0.98 1.08)	0.31**	0.85 (0.64 1.13)	1.00	1.09 (0.89 1.34)	1.02 (0.73 1.42)
	HR (95% CI)^f^	1.02 (0.97 1.08)	0.34**	0.86 (0.65 1.14)	1.00	1.09 (0.88 1.34)	1.02 (0.73 1.42)
Stomach	Cases	356		36	164	121	35
	HR (95% CI)*	**1.14 (1.08 1.20)**	**< 0.001**	**0.61 (0.43 0.88)**	1.00	**1.34 (1.06 1.70)**	**1.55 (1.08 2.24)**
	HR (95% CI)†	**1.06 (1.001 1.13)**	**0.045**	**0.66 (0.45 0.97)**	1.00	1.12 (0.87 1.44)	1.03 (0.69 1.53)
	HR (95% CI)	**1.06 (1.001 1.13)**	**0.045**	**0.66 (0.45 0.97)**	1.00	1.12 (0.87 1.44)	1.03 (0.69 1.53)
Oesophagus and stomach	Cases	891		105	405	297	84
	HR (95% CI)*	**1.12 (1.08 1.15)**	**< 0.001**	**0.73 (0.59 0.90)**	1.00	**1.33 (1.15 1.55)**	**1.50 (1.19 1.90)**
	HR (95% CI)†	**1.04 (1.005 1.09)**	**0.03**	**0.78 (0.62 0.98)**	1.00	1.12 (0.95 1.31)	1.04 (0.81 1.34)
	HR (95% CI)	**1.04 (1.005 1.09)**	**0.03**	**0.78 (0.62 0.98)**	1.00	1.12 (0.95 1.31)	1.04 (0.81 1.34)
Hepatobiliary tract	Cases	456		74	203	130	49
	HR (95% CI)*	**1.08 (1.03 1.14)**	**0.002**	1.02 (0.78 1.33)	1.00	1.15 (0.93 1.44)	**1.77 (1.30 2.43)**
	HR (95% CI)†	1.01 (0.96 1.07)	0.62	1.08 (0.82 1.43)	1.00	0.98 (0.78 1.24)	1.26 (0.90 1.77)
	HR (95% CI)	1.01 (0.96 1.07)	0.62	1.08 (0.82 1.43)	1.00	0.98 (0.78 1.24)	1.26 (0.90 1.77)
Pancreatic	Cases	615		97	283	187	48
	HR (95% CI)*	**1.07 (1.02 1.11)**	**0.004**	0.96 (0.76 1.21)	1.00	1.19 (0.99 1.43)	1.25 (0.92 1.70)
	HR (95% CI)†	1.04 (0.99 1.09)	0.15	0.99 (0.78 1.27)	1.00	1.12 (0.92 1.36)	1.07 (0.77 1.49)
	HR (95% CI)^d^	1.03 (0.98 1.08)	0.20	0.996 (0.78 1.27)	1.00	1.11 (0.92 1.36)	1.03 (0.74 1.44)
Kidney	Cases	779		113	390	206	70
	HR (95% CI)*	**1.06 (1.02 1.10)**	**0.007**	**0.79 (0.64 0.98)**	1.00	0.99 (0.83 1.17)	**1.37 (1.06 1.76)**
	HR (95% CI)†	0.996 (0.95 1.04)	0.86**	0.92 (0.74 1.14)	1.00	0.88 (0.74 1.05)	1.08 (0.82 1.42)
	HR (95% CI)	0.996 (0.95 1.04)	0.86**	0.92 (0.74 1.14)	1.00	0.88 (0.74 1.05)	1.08 (0.82 1.42)
Bladder	Cases	677		92	295	221	69
	HR (95% CI)*	**1.10 (1.05 1.14)**	**< 0.001**	0.90 (0.71 1.14)	1.00	**1.32 (1.10 1.57)**	**1.62 (1.25 2.11)**
	HR (95% CI)†	1.04 (0.99 1.09)	0.13**	1.04 (0.81 1.32)	1.00	**1.21 (1.002 1.45)**	1.29 (0.97 1.73)
	HR (95% CI)	1.04 (0.99 1.09)	0.13**	1.04 (0.81 1.32)	1.00	**1.21 (1.002 1.45)**	1.29 (0.97 1.73)
Colorectal	Cases	3358		538	1643	936	241
	HR (95% CI)*	**1.03 (1.01 1.05)**	0.001**	**0.90 (0.82 0.99)**	1.00	1.05 (0.97 1.14)	1.11 (0.97 1.28)
	HR (95% CI)†	1.02 (0.999 1.04)	0.07**	0.93 (0.84 1.03)	1.00	1.03 (0.94 1.12)	1.05 (0.90 1.22)
	HR (95% CI)^e, g, f (males)^	1.02 (0.995 1.04)	0.13**	0.93 (0.83 1.03)	1.00	1.02 (0.93 1.11)	1.03 (0.88 1.20)
Colon	Cases	2155		329	1041	614	171
	HR (95% CI)*	**1.05 (1.02 1.08)**	< 0.001**	**0.87 (0.77 0.99)**	1.00	**1.08 (0.98 1.19)**	**1.24 (1.05 1.45)**
	HR (95% CI)†	**1.04 (1.01 1.07)**	0.007**	0.92 (0.81 1.05)	1.00	1.05 (0.94 1.17)	**1.19 (1.003 1.42)**
	HR (95% CI)^e, g, f (males)^	**1.04 (1.01 1.06)**	0.02**	0.93 (0.81 1.06)	1.00	1.05 (0.94 1.16)	1.17 (0.98 1.41)
Rectum	Cases	1127		196	556	307	68
	HR (95% CI)*	1.01 (0.98 1.05)	0.53**	0.96 (0.81 1.13)	1.00	1.04 (0.90 1.20)	0.94 (0.73121)
	HR (95% CI)†	0.996 (0.96 1.04)	0.84**	0.98 (0.82 1.16)	1.00	1.04 (0.89 1.20)	0.84 (0.63 1.11)
	HR (95% CI)^e, g^	0.99 (0.95 1.03)	0.67	0.96 (0.81 1.14)	1.00	1.01 (0.87 1.18)	0.82 (0.62 1.10)
Brain tumours	Cases	463		82	237	114	30
	HR (95% CI)*	1.03 (0.98 1.08)	0.29	0.92 (0.72 1.19)	1.00	0.93 (0.74 1.16)	1.003 (0.69 1.47)
	HR (95% CI)†	1.04 (0.98 1.10)	0.20	0.87 (0.66 1.13)	1.00	0.92 (0.73 1.17)	0.96 (0.63 1.46)
	HR (95% CI)	1.04 (0.98 1.10)	0.20	0.87 (0.66 1.13)	1.00	0.92 (0.73 1.17)	0.96 (0.63 1.46)
Thyroid	Cases	242		48	124	57	13
	HR (95% CI)*	0.99 (0.91 1.07)	0.75	0.95 (0.68 1.32)	1.00	0.97 (0.70 1.33)	0.97 (0.55 1.73)
	HR (95% CI)†	1.001 (0.92 1.09)	0.98	0.92 (0.65 1.30)	1.00	0.93 (0.66 1.32)	1.14 (0.63 2.06)
	HR (95% CI)	1.001 (0.92 1.09)	0.98	0.92 (0.65 1.30)	1.00	0.93 (0.66 1.32)	1.14 (0.63 2.06)
Haematological malignancies	Cases	2468		438	1208	652	170
	HR (95% CI)*	1.01 (0.98 1.03)	0.52	0.995 (0.89 1.11)	1.00	0.99 (0.90 1.09)	1.06 (0.91 1.25)
	HR (95% CI)†	1.002 (0.98 1.03)	0.89	0.97 (0.87 1.09)	1.00	0.97 (0.88 1.08)	0.97 (0.82 1.16)
	HR (95% CI)	1.002 (0.98 1.03)	0.89	0.97 (0.87 1.09)	1.00	0.97 (0.88 1.08)	0.97 (0.82 1.16)
Non-Hodgkin’s lymphoma	Cases	1193		197	586	337	73
	HR (95% CI)*	1.01 (0.98 1.05)	0.44	0.92 (0.78 1.08)	1.00	1.06 (0.93 1.21)	0.95 (0.74 1.21)
	HR (95% CI)†	1.01 (0.98 1.05)	0.48	0.89 (0.75 1.05)	1.00	1.08 (0.93 1.24)	0.85 (0.65 1.11)
	HR (95% CI)	1.01 (0.98 1.05)	0.48	0.89 (0.75 1.05)	1.00	1.08 (0.93 1.24)	0.85 (0.65 1.11)

*Models adjusted for age and sex (total observations = 470,578)

†Models adjusted for age, sex, ethnicity (white/other), deprivation index (quintiles), education (University degree, A-levels/HNC/HND/NVQ, GCSE/O-level/CSE, OTHER, None), fruit and vegetable intake (< 5 portions/day, ≥5 portions/day), BMI (kg/m2), height (m), smoking status (never, former light smoker [< 20 pack-years], former heavy smoker [≥20 pack-years], current light smoker [< 20 pack-years], current heavy smoker [≥20 pack-years]) and alcohol intake (never, former, current [<once/week], current [≥once/week])

^a^Additional site-specific covariates in the final model include use of sun/UV protection (Never/rarely/sometimes; most of the time/always; do not go out in sunshine)

^b^Additional site-specific covariates in the final model include HRT use (ever used/never used), oral contraceptive use (ever used/never used), number of live births (0, 1, 2, 3+ live births), age at menarche (early menarche [< 12 years], menarche at 12–14 years, late menarche [≥15 years]), age at menopause (< 40 years, 40–44 years, 45–49 years, 50–54 years, 55–59 years, 60–64 years, ≥65 years, not had menopause/unsure), hysterectomy status (had hysterectomy, not had hysterectomy/unsure)

^c^Additional site-specific covariates in the final model include HRT use (ever used/never used), oral contraceptive use (ever used/never used), number of live births (0, 1, 2, 3+ live births), age at menarche (early menarche [< 12 years], menarche at 12–14 years, late menarche [≥15 years]), age at menopause (< 40 years, 40–44 years, 45–49 years, 50–54 years, 55–59 years, 60–64 years, ≥65 years, not had menopause/unsure), hysterectomy status (had hysterectomy, not had hysterectomy/unsure)

^d^Additional site-specific covariates in the final model include diabetes at baseline (yes/no)

^e^Additional site-specific covariates in the final model include diabetes at baseline (yes/no), aspirin use (regular use/non-regular use or no use), HRT use (ever used/never used; females only), red meat intake (portion/week), processed meat intake (portion/week)

^f^Final model also adjusted for waist-hip ratio (> 94 cm in men, > 80 cm in women)

^f(males)^For cancer sites which were adjusted for different sets of covariates for males and females (colorectal, colon, rectum), this indicates that the final model for male participants was also adjusted for waist-hip ratio (> 94 cm in men)

^g^Results for males and females combined using meta-analysis as covariates are different

^h^Final model also adjusted for family history of cancer (mother/father/sibling had cancer, no family history)

**Schoenfeld test indicated potential violation of the proportional hazards assumption (*p* < 0.05)

Participants who reported ≤1 h/day of TV viewing time had a lower risk of lung cancer (HR 0.85, 95% CI: 0.73, 0.997), breast (female only) cancer (HR 0.92, 95% CI: 0.85, 0.996), stomach cancer (HR 0.66, 95% CI: 0.45, 0.97), and oesophagus and stomach cancer (HR 0.78, 95% CI: 0.62, 0.98) compared to participants who reported 1- ≤ 3 h/day of TV viewing time.

After excluding cancers diagnosed within the first two years following baseline, all associations were attenuated except those for oesophagus and stomach cancers, and colon cancers (Table 3). Whilst the results of the Schoenfeld residual tests indicated that some of our models may not have been in line with the proportional hazards assumption, our visual inspection of log-log plots and Schoenfeld residual plots showed no serious violations. Therefore, we proceeded with the analyses as planned. Further, we have included our analyses by both continuous and categorical variables side-by-side in the main outcomes tables which facilitated stratification by categories of potentially violating variables.

**Table 3:** Results of Cox proportional hazards analyses investigating the association between self-report daily TV viewing time and cancer incidence (excluding cancers diagnosed within the first 2 years following baseline)

		1 h increase in TV viewing time	*p*-value	≤1 h	1- ≤ 3 h (reference)	3- ≤ 5 h	> 5 h
Skin, melanoma	Cases	1192		222	613	299	58
	HR (95% CI)^a^	1.02(0.98,1.06)	0.39**	0.93(0.80,1.09)	1.00	1.04(0.90,1.20)	1.004(0.76,1.33)
Oropharyngeal	Cases	410		69	197	105	39
	HR (95% CI)	1.04(0.99,1.10)	0.12	0.90(0.68,1.19)	1.00	1.02(0.80,1.30)	1.27(0.89,1.84)
Lung	Cases	1461		165	638	467	191
	HR (95% CI)^h^	1.02(0.99,1.05)	0.21**	0.87(0.73,1.03)	1.00	0.97(0.86,1.10)	1.10(0.93,1.30)
Breast (female only)	Cases	3288		657	1724	756	151
	HR (95% CI)^b, h^	1.004(0.98,1.03)	0.76**	**0.90(0.82,0.99)**	1.00	0.96(0.87,1.04)	0.94(0.79,1.11)
Uterus	Cases	567		102	270	165	30
	HR (95% CI)^c^	0.98(0.93,1.04)	0.56**	1.05(0.83,1.33)	1.00	1.06(0.87,1.29)	0.75(0.50,1.10)
Ovary	Cases	404		69	204	109	22
	HR (95% CI)	1.02(0.95,1.09)	0.60	0.88(0.67,1.16)	1.00	1.01(0.80,1.29)	0.93(0.59,1.46)
Prostate	Cases	4235		804	2127	1069	235
	HR (95% CI)^h^	0.99(0.97,1.01)	0.34**	1.02(0.94,1.10)	1.00	0.98(0.91,1.06)	0.94(0.82,1.08)
Oesophagus	Cases	392		46	182	125	39
	HR (95% CI)^f^	1.04(0.98,1.10)	0.20**	0.79(0.57,1.10)	1.00	1.08(0.86,1.37)	1.11(0.77,1.60)
Stomach	Cases	250		24	125	78	23
	HR (95% CI)	1.06(0.99,1.14)	0.09	**0.57(0.37,0.89)**	1.00	0.996(0.74,1.33)	0.96(0.60,1.53)
Oesophagus and stomach	Cases	638		70	303	203	62
	HR (95% CI)	**1.05(1.011.10)**	0.03**	**0.71(0.54,0.92)**	1.00	1.07(0.89,1.28)	1.07(0.80,1.42)
Hepatobiliary tract	Cases	348		51	156	100	41
	HR (95% CI)	1.03(0.97,1.10)	0.29	0.9999(0.72,1.38)	1.00	1.001(0.77,1.29)	1.36(0.94,1.95)
Pancreatic	Cases	463		75	215	140	33
	HR (95% CI)^d^	1.01(0.95,1.06)	0.85	1.02(0.78,1.33)	1.00	1.09(0.88,1.36)	0.91(0.62,1.34)
Kidney	Cases	583		90	280	161	52
	HR (95% CI)	0.99(0.94,1.04)	0.75	1.02(0.80,1.30)	1.00	0.95(0.77,1.15)	1.11(0.81,1.51)
Bladder	Cases	461		57	208	155	41
	HR (95% CI)	1.03(0.98,1.09)	0.25**	0.88(0.65,1.19)	1.00	1.19(0.96,1.47)	1.10(0.77,1.55)
Colorectal	Cases	2281		383	1118	621	159
	HR (95% CI)^e, g, f (males)^	1.03(0.998,1.05)	0.07**	0.95(0.85,1.07)	1.00	1.03(0.93,1.14)	1.10(0.93,1.31)
Colon	Cases	1478		246	712	407	113
	HR (95% CI)^e, g, f (males)^	**1.04(1.01,1.07)**	0.02**	0.97(0.84,1.13)	1.00	1.05(0.92,1.19)	1.22(0.99,1.50)
Rectum	Cases	754		134	373	201	46
	HR (95% CI)^e, g^	1.000(0.96,1.05)	0.995	0.98(0.80,1.20)	1.00	1.03(0.87,1.23)	0.99(0.72,1.36)
Brain tumours	Cases	333		58	178	80	17
	HR (95% CI)	1.03(0.96,1.11)	0.38	0.85(0.62,1.15)	1.00	0.89(0.68,1.17)	0.81(0.48,1.35)
Thyroid	Cases	161		32	85	35	9
	HR (95% CI)	0.99(0.89,1.09)	0.78	0.91(0.60,1.38)	1.00	0.88(0.59,1.32)	0.998(0.49,2.03)
Haematological malignancies	Cases	1786		315	888	470	113
	HR (95% CI)	1.003(0.97,1.03)	0.86**	0.96(0.84,1.10)	1.00	0.98(0.87,1.10)	0.95(0.78,1.17)
Non-Hodgkin’s lymphoma	Cases	886		138	431	254	43
	HR (95% CI)	1.02(0.98,1.07)	0.26	0.84(0.69,1.02)	1.00	1.12(0.95,1.31)	0.78(0.57,1.08)

^a^Additional site-specific covariates in the final model include use of sun/UV protection (Never/rarely/sometimes; most of the time/always; do not go out in sunshine)

^b^Additional site-specific covariates in the final model include HRT use (ever used/never used), oral contraceptive use (ever used/never used), number of live births (0, 1, 2, 3+ live births), age at menarche (early menarche [< 12 years], menarche at 12–14 years, late menarche [≥15 years]), age at menopause (< 40 years, 40–44 years, 45–49 years, 50–54 years, 55–59 years, 60–64 years, ≥65 years, not had menopause/unsure), hysterectomy status (had hysterectomy, not had hysterectomy/unsure)

^c^Additional site-specific covariates in the final model include HRT use (ever used/never used), oral contraceptive use (ever used/never used), number of live births (0, 1, 2, 3+ live births), age at menarche (early menarche [< 12 years], menarche at 12–14 years, late menarche [≥15 years]), age at menopause (< 40 years, 40–44 years, 45–49 years, 50–54 years, 55–59 years, 60–64 years, ≥65 years, not had menopause/unsure), hysterectomy status (had hysterectomy, not had hysterectomy/unsure)

^d^Additional site-specific covariates in the final model include diabetes at baseline (yes/no)

^e^Additional site-specific covariates in the final model include diabetes at baseline (yes/no), aspirin use (regular use/non-regular use or no use), HRT use (ever used/never used; females only), red meat intake (portion/week), processed meat intake (portion/week)

^f^Final model also adjusted for waist-hip ratio (> 94 cm in men, > 80 cm in women)

^f(males)^For cancer sites which were adjusted for different sets of covariates for males and females (colorectal, colon, rectum), this indicates that the final model for male participants was also adjusted for waist-hip ratio (> 94 cm in men)

^g^Results for males and females combined using meta-analysis as covariates are different

^h^Final model also adjusted for family history of cancer (mother/father/sibling had cancer, no family history)

**Schoenfeld test indicated potential violation of the proportional hazards assumption (*p* < 0.05)

Subgroup analyses and tests of effect modification (supplement 1, Tables 1.2–1.9) showed that HR estimates between daily TV viewing time and lung cancer risk differed according to an area-based measure of deprivation (p for interaction =0.004). For example, for participants who ranked in the top 20% least deprived (i.e. most affluent) area of residence, a 1-h increase in daily TV viewing time was associated with a lower risk of lung cancer (HR 0.87, 95% CI: 0.79, 0.95), whereas risk was increased (HR 1.06, 95% CI: 1.004, 1.12) or null (HR 1.02, 95% CI: 0.99, 1.06) for individuals residing in the top 40 and 20% most deprived areas, respectively (Supplementary Table 1.4).

The results of other subgroup analyses were mostly non-significant according to our threshold (p for interaction > 0.01), however some differential associations were evident when comparing magnitudes of hazard ratios or precision of confidence intervals. For example, in analyses stratified by sex (Supplementary Table 1.2), 1 h increases in TV viewing time were associated with significant increased oropharyngeal, lung and colon cancer risk in males only, and increased kidney cancer risk in females only. No clear dose-response associations were observed between increased TV viewing and cancer risk across age groups (Supplementary Table 1.3), or categories of smoking status (Supplementary Table 1.5). The increased risk for oesophago-gastric cancer with each 1 h increase in daily TV viewing did appear to be strongest in never smokers (HR 1.11, 95% CI: 1.04, 1.19) although the p for interaction wasn’t significant (*p* = 0.27).

Supplementary Tables 1.6, 1.7, 1.8 illustrate results from analyses stratified by body composition, physical activity levels, or a combination of these (to reflect the ‘fat but fit’ hypothesis). The magnitude of increased oropharyngeal and oesophago-gastric cancer risks for each 1 h increase in daily TV viewing were small, but strongest in individuals who were non-obese (Supplementary Table 1.6), but did not differ across categories of physical activity (Supplementary Table 1.7), although tests for interaction were not significant. Significant interactions were observed according to physical activity level for rectum cancers amongst males (p for interaction = 0.004). According to strata of combined body fat percentage and physical activity level categories, the associations between daily TV viewing and risk of lung cancer (p for interaction = 0.003) and haematological malignancies (p for interaction = 0.0004), as shown in Supplementary Table 1.8. This is difficult to interpret, as the risk estimates do not differ considerably between categories, however are slightly lower in individuals with high physical activity but high body fat percentages. Finally, menopausal status did not alter the null associations between daily TV viewing time and risk of female cancers (Supplementary Table 1.9). Overall, these stratified analyses generate some interesting hypotheses, but results should be interpreted with caution due to small numbers and multiple testing.

### Results of partition models and isotemporal substitution models

Partition models showed there was an association between a 1-h increase in daily TV viewing time and a higher risk of oropharyngeal cancer (HR 1.11, 95% CI: 1.05, 1.17) and lung cancer (HR 1.04, 95% CI: 1.01, 1.07), when holding daily time spent in moderate-intensity physical activity, vigorous-intensity physical activity and walking constant. There was an association between a 1-h increase in daily time spent in moderate-intensity physical activity and a lower risk of breast (female only) cancer (HR 0.91, 95% CI: 0.86, 0.96), and colon cancer (HR 0.89, 95% CI: 0.81, 0.97) when holding daily TV viewing time, and time spent in vigorous-intensity physical activity and walking constant (supplement 1, Table 1.1).

Isotemporal substitution models showed there was an association between replacing 1-h of daily TV viewing time with 1-h of moderate-intensity physical activity and a lower risk of breast (female only) cancer (HR 0.90, 95% CI: 0.85, 0.96), colorectal cancer (HR 0.92, 95% CI: 0.86, 0.99) and colon cancer (HR 0.87, 95% CI: 0.79, 0.95), when holding time spent in vigorous-intensity physical activity and walking constant. There was an association between replacing 1-h of daily TV viewing time with 1-h of walking and a lower risk of oropharyngeal cancer (HR 0.79, 95% CI: 0.67, 0.92), and lung cancer (HR 0.89, 95% CI: 0.82, 0.97) when holding time spent in moderate- and vigorous-intensity physical activity constant (Table 4).

**Table 4:** Results of isotemporal substitution models showing the impact on cancer incidence of replacing a 1-h of total daily TV viewing time with the same amount of daily moderate activity, daily vigorous activity or daily walking time, holding the other activities constant

	1-h increase in daily moderate activity	1-h increase in daily vigorous activity	1-h increase in daily walking time
	HR (95% CI)	HR (95% CI)	HR (95% CI)
**Skin melanoma** [cases = 1256]^a^	0.98 (0.89, 1.09)	0.97 (0.81, 1.17)	1.03 (0.94, 1.12)
**Oropharyngeal** [cases = 411]	0.91 (0.77, 1.08)	0.86 (0.63, 1.18)	**0.79 (0.67, 0.92)**
**Lung** [cases = 1355]^h^	1.0003 (0.92, 1.09)	0.84 (0.71, 1.004)	**0.89 (0.82, 0.97)**
**Breast (female only)** [cases = 3454]^b, h^	**0.90 (0.85, 0.96)**	1.02 (0.89, 1.16)	0.99 (0.94, 1.05)
**Uterus** [cases = 570]^c^	1.001 (0.86, 1.17)	1.05 (0.76, 1.46)	0.99 (0.86, 1.13)
**Ovary** [cases = 405]	1.09 (0.93, 1.28)	1.12 (0.81, 1.55)	0.97 (0.83, 1.13)
**Prostate** [cases = 4629]^h^	1.01 (0.96, 1.06)	1.05 (0.97, 1.15)	0.9997 (0.95, 1.05)
**Oesophagus** [cases = 386]^f^	1.09 (0.93, 1.28)	1.06 (0.80, 1.42)	0.91 (0.77, 1.06)
**Stomach** [cases = 264]	1.06 (0.87, 1.29)	0.77 (0.52, 1.15)	0.91 (0.76, 1.10)
**Oesophagus and stomach** [cases = 644]	1.08 (0.95, 1.22)	0.94 (0.74, 1.18)	0.90 (0.80, 1.02)
**Hepatobiliary tract** [cases = 331]	0.84 (0.69, 1.02)	1.01 (0.71, 1.43)	1.03 (0.87, 1.21)
**Pancreatic** [cases = 467]^d^	1.07 (0.92, 1.24)	0.92 (0.69, 1.23)	0.95 (0.82, 1.09)
**Kidney** [cases = 559]	1.01 (0.88, 1.17)	1.12 (0.87, 1.44)	0.95 (0.83, 1.09)
**Bladder** [cases = 502]	0.98 (0.85, 1.13)	0.83 (0.62, 1.09)	1.03 (0.90, 1.17)
**Colorectal** [cases = 2405]^e, g, f (males)^	**0.92 (0.86, 0.99)**	0.997 (0.87, 1.14)	1.01 (0.95, 1.08)
**Colon** [cases = 1530]^e, g, f (males)^	**0.87 (0.79, 0.95)**	0.96 (0.81, 1.14)	1.001 (0.92, 1.09)
**Rectum** [cases = 821]^e, g^	0.99 (0.88, 1.12)	1.06 (0.86, 1.30)	1.01 (0.90, 1.12)
**Brain tumours** [cases = 345]	0.85 (0.70, 1.03)	0.85 (0.59, 1.23)	1.04 (0.88, 1.23)
**Thyroid** [cases = 181]	0.94 (0.72, 1.23)	0.80 (0.46, 1.40)	1.07 (0.85, 1.35)
**Haematological malignancies** [cases = 1794]	0.98 (0.90, 1.06)	1.07 (0.93, 1.24)	0.99 (0.92, 1.07)
**Non-Hodgkin’s lymphoma** [cases = 864]	0.99 (0.88, 1.11)	1.07 (0.87, 1.32)	0.95 (0.85, 1.06)

All models were adjusted for age, sex, ethnicity (white/other), deprivation index (quintiles), education (University degree, A-levels/HNC/HND/NVQ, GCSE/O-level/CSE, OTHER, None), fruit and vegetable intake (< 5 portions/day, ≥5 portions/day), BMI (kg/m2), height (m), smoking status (never, former light smoker [< 20 pack-years], former heavy smoker [≥20 pack-years], current light smoker [< 20 pack-years], current heavy smoker [≥20 pack-years]) and alcohol intake (never, former, current [<once/week], current [≥once/week])

^a^Additional site-specific covariates in the final model include use of sun/UV protection (Never/rarely/sometimes; most of the time/always; do not go out in sunshine)

^b^Additional site-specific covariates in the final model include HRT use (ever used/never used), oral contraceptive use (ever used/never used), number of live births (0, 1, 2, 3+ live births), age at menarche (early menarche [< 12 years], menarche at 12–14 years, late menarche [≥15 years]), age at menopause (< 40 years, 40–44 years, 45–49 years, 50–54 years, 55–59 years, 60–64 years, ≥65 years, not had menopause/unsure), hysterectomy status (had hysterectomy, not had hysterectomy/unsure)

^c^Additional site-specific covariates in the final model include HRT use (ever used/never used), oral contraceptive use (ever used/never used), number of live births (0, 1, 2, 3+ live births), age at menarche (early menarche [< 12 years], menarche at 12–14 years, late menarche [≥15 years]), age at menopause (< 40 years, 40–44 years, 45–49 years, 50–54 years, 55–59 years, 60–64 years, ≥65 years, not had menopause/unsure), hysterectomy status (had hysterectomy, not had hysterectomy/unsure)

^d^Additional site-specific covariates in the final model include diabetes at baseline (yes/no)

^e^Additional site-specific covariates in the final model include diabetes at baseline (yes/no), aspirin use (regular use/non-regular use or no use), HRT use (ever used/never used; females only), red meat intake (portion/week), processed meat intake (portion/week)

^f^Final model also adjusted for waist-hip ratio (> 94 cm in men, > 80 cm in women)

^f(males)^For cancer sites which were adjusted for different sets of covariates for males and females (colorectal, colon, rectum), this indicates that the final model for male participants was also adjusted for waist-hip ratio (> 94 cm in men)

^g^Results for males and females combined using meta-analysis as covariates are different

^h^Final model also adjusted for family history of cancer (mother/father/sibling had cancer, no family history)

### Association of site-specific cancer risk and daily recreational computer time

Table 5 shows the association between a 1-h increase in daily recreational computer time and site-specific cancer risk. A 1-h increase in daily recreational computer time was associated with lower risk of oropharyngeal cancer (HR 0.93, 95% CI: 0.87, 0.998). The categorical analysis showed that participants who reported that they spent no hours using computers had a higher risk of oropharyngeal cancer (HR 1.27, 95% CI: 1.03, 1.56), and ovary cancer (HR 1.23, 95% CI: 1.01, 1.50) compared to participants who reported ≤1 h of daily recreational computer time.

**Table 5:** Results of Cox proportional hazards analyses investigating the association between self-report daily computer use time and cancer incidence

		1 h increase in computer use time	*p*-value	None	≤1 h (reference)	1- ≤ 3 h	> 3 h
Person-years		3,498,487		969,721	1,744,785	582,168	201,813
Skin, melanoma	Cases	1621		404	852	276	89
	HR (95% CI)*	1.01 (0.98 1.05)	0.43**	**0.77 (0.68 0.86)**	1.00	0.90 (0.79 1.04)	0.92 (0.74 1.14)
	HR (95% CI)†	1.01 (0.97 1.05)	0.72	0.90 (0.79 1.02)	1.00	0.97 (0.85 1.12)	0.99 (0.79 1.24)
	HR (95% CI)^a^	1.01 (0.97 1.05)	0.73	0.91 (0.80 1.03)	1.00	0.98 (0.85 1.12)	0.996 (0.79 1.25)
Oropharyngeal	Cases	561		209	239	88	25
	HR (95% CI)*	**0.90 (0.84 0.97)**	**0.004**	**1.56 (1.29 1.88)**	1.00	0.97 (0.76 1.23)	0.81 (0.54 1.23)
	HR (95% CI)†	**0.93 (0.87 0.998)**	**0.04**	**1.27 (1.03 1.56)**	1.00	0.91 (0.71 1.17)	0.77 (0.51 1.17)
	HR (95% CI)	**0.93 (0.87 0.998)**	**0.04**	**1.27 (1.03 1.56)**	1.00	0.91 (0.71 1.17)	0.77 (0.51 1.17)
Lung	Cases	2040		894	700	316	130
	HR (95% CI)*	0.97 (0.93 1.003)	0.08**	**1.84 (1.66 2.03)**	1.00	**1.17 (1.03 1.34)**	**1.68 (1.39 2.03)**
	HR (95% CI)†	1.02 (0.99 1.06)	0.16**	1.10 (0.99 1.23)	1.00	1.01 (0.88 1.16)	**1.33 (1.10 1.62)**
	HR (95% CI)^h^	1.02 (0.99 1.06)	0.16**	1.11 (0.99 1.24)	1.00	0.996 (0.87 1.15)	**1.36 (1.12 1.65)**
Breast (female only)	Cases	5650		1728	2931	762	229
	HR (95% CI)*	1.01 (0.99 1.03)	0.27**	**0.93 (0.88 0.99)**	1.00	1.02 (0.94 1.10)	0.997 (0.87 1.14)
	HR (95% CI)†	1.003 (0.98 1.03)	0.83**	0.97 (0.91 1.04)	1.00	0.999 (0.92 1.08)	1.001 (0.87 1.15)
	HR (95% CI)^b, f, h^	1.01 (0.98 1.03)	0.57**	0.96 (0.90 1.04)	1.00	0.998 (0.91 1.09)	1.03 (0.89 1.20)
Uterus	Cases	863		315	389	127	32
	HR (95% CI)*	1.01 (0.95 1.07)	0.74	**1.18 (1.02 1.38)**	1.00	**1.25 (1.03 1.53)**	1.12 (0.78 1.60)
	HR (95% CI)†	0.96 (0.90 1.02)	0.20	1.18 (0.999 1.39)	1.00	1.04 (0.84 1.28)	0.91 (0.63 1.33)
	HR (95% CI)^c^	0.98 (0.92 1.04)	0.47	1.16 (0.98 1.37)	1.00	1.08 (0.87 1.35)	0.95 (0.64 1.40)
Ovary	Cases	567		211	266	63	27
	HR (95% CI)*	0.98 (0.91 1.05)	0.51	1.16 (0.96 1.39)	1.00	0.91 (0.69 1.19)	1.36 (0.91 2.02)
	HR (95% CI)†	0.96 (0.89 1.04)	0.36	**1.23 (1.01 1.50)**	1.00	0.91 (0.69 1.21)	1.36 (0.91 2.04)
	HR (95% CI)	0.96 (0.89 1.04)	0.36	**1.23 (1.01 1.50)**	1.00	0.91 (0.69 1.21)	1.36 (0.91 2.04)
Prostate	Cases	5933		1543	2699	1298	393
	HR (95% CI)*	1.005 (0.99 1.02)	0.61**	**0.91 (0.85 0.97)**	1.00	0.97 (0.91 1.03)	0.97 (0.87 1.07)
	HR (95% CI)†	0.998 (0.98 1.02)	0.85**	0.98 (0.92 1.06)	1.00	0.99 (0.93 1.06)	0.9998 (0.90 1.12)
	HR (95% CI)^h^	0.997 (0.98 1.02)	0.73**	0.99 (0.92 1.06)	1.00	0.99 (0.93 1.06)	0.99 (0.89 1.11)
Oesophagus	Cases	530		174	221	108	27
	HR (95% CI)*	0.97 (0.90 1.03)	0.32	1.20 (0.98 1.47)	1.00	1.13 (0.90 1.42)	0.94 (0.63 1.41)
	HR (95% CI)†	0.97 (0.90 1.04)	0.37	0.99 (0.79 1.23)	1.00	1.02 (0.81 1.30)	0.78 (0.51 1.19)
	HR (95% CI)^f^	0.97 (0.90 1.04)	0.35	0.99 (0.79 1.23)	1.00	1.02 (0.80 1.30)	0.77 (0.51 1.18)
Stomach	Cases	349		133	133	61	22
	HR (95% CI)*	0.98 (0.90 1.06)	0.60	**1.50 (1.18 1.92)**	1.00	1.09 (0.80 1.47)	1.30 (0.83 2.05)
	HR (95% CI)†	0.98 (0.91 1.07)	0.71	1.16 (0.88 1.51)	1.00	0.98 (0.72 1.35)	1.04 (0.64 1.69)
	HR (95% CI)	0.98 (0.91 1.07)	0.71	1.16 (0.88 1.51)	1.00	0.98 (0.72 1.35)	1.04 (0.64 1.69)
Oesophagus and stomach	Cases	873		305	352	168	48
	HR (95% CI)*	0.97 (0.92 1.02)	0.25	**1.31 (1.12 1.53)**	1.00	1.11 (0.93 1.34)	1.06 (0.79 1.44)
	HR (95% CI)†	0.97 (0.92 1.03)	0.33	1.05 (0.89 1.25)	1.00	1.01 (0.83 1.22)	0.86 (0.62 1.19)
	HR (95% CI)^f^	0.97 (0.92 1.03)	0.32	1.05 (0.89 1.25)	1.00	1.01 (0.83 1.22)	0.86 (0.62 1.19)
Hepatobiliary tract	Cases	451		170	168	91	22
	HR (95% CI)*	0.97 (0.90 1.04)	0.42	**1.49 (1.20 1.85)**	1.00	**1.38 (1.07 1.79)**	1.14 (0.73 1.77)
	HR (95% CI)†	0.99 (0.92 1.06)	0.74	1.21 (0.95 1.53)	1.00	1.30 (0.997 1.69)	1.02 (0.65 1.61)
	HR (95% CI)	0.99 (0.92 1.06)	0.74	1.21 (0.95 1.53)	1.00	1.30 (0.997 1.69)	1.02 (0.65 1.61)
Pancreatic	Cases	606		189	276	114	27
	HR (95% CI)*	0.98 (0.92 1.05)	0.62	1.01 (0.83 1.21)	1.00	1.07 (0.86 1.34)	0.87 (0.59 1.29)
	HR (95% CI)†	0.98 (0.92 1.05)	0.62	0.90 (0.73 1.11)	1.00	1.05 (0.84 1.31)	0.76 (0.50 1.15)
	HR (95% CI)^d^	0.98 (0.92 1.05)	0.61	0.90 (0.73 1.10)	1.00	1.05 (0.84 1.31)	0.76 (0.50 1.14)
Kidney	Cases	783		251	333	149	50
	HR (95% CI)*	1.01 (0.96 1.07)	0.60	1.17 (0.995 1.39)	1.00	1.12 (0.92 1.36)	1.23 (0.91 1.66)
	HR (95% CI)†	1.02 (0.97 1.08)	0.39**	1.04 (0.87 1.25)	1.00	1.05 (0.86 1.29)	1.19 (0.88 1.61)
	HR (95% CI)	1.02 (0.97 1.08)	0.39**	1.04 (0.87 1.25)	1.00	1.05 (0.86 1.29)	1.19 (0.88 1.61)
Bladder	Cases	670		227	271	142	30
	HR (95% CI)*	0.98 (0.93 1.04)	0.54	**1.22 (1.02 1.46)**	1.00	1.18 (0.96 1.44)	0.85 (0.59 1.25)
	HR (95% CI)†	0.97 (0.92 1.04)	0.41**	1.09 (0.89 1.32)	1.00	1.08 (0.87 1.33)	0.76 (0.51 1.13)
	HR (95% CI)	0.97 (0.92 1.04)	0.41**	1.09 (0.89 1.32)	1.00	1.08 (0.87 1.33)	0.76 (0.51 1.13)
Colorectal	Cases	3312		1059	1512	556	185
	HR (95% CI)*	0.99 (0.96 1.02)	0.45**	1.07 (0.99 1.16)	1.00	0.95 (0.87 1.05)	1.05 (0.90 1.23)
	HR (95% CI)†	0.99 (0.96 1.01)	0.31**	1.08 (0.99 1.18)	1.00	0.95 (0.86 1.05)	1.03 (0.88 1.21)
	HR (95% CI)^e, g, f (males)^	0.98 (0.96 1.01)	0.28**	1.06 (0.97 1.16)	1.00	0.95 (0.86 1.05)	1.02 (0.87 1.20)
Colon	Cases	2124		681	980	348	115
	HR (95% CI)*	0.99 (0.96 1.03)	0.63**	1.04 (0.94 1.15)	1.00	0.94 (0.83 1.06)	1.04 (0.86 1.26)
	HR (95% CI)†	0.99 (0.95 1.02)	0.50**	1.04 (0.93 1.15)	1.00	0.93 (0.82 1.06)	1.02 (0.83 1.24)
	HR (95% CI)^e, g, f (males)^	0.99 (0.95 1.02)	0.42**	1.03 (0.92 1.14)	1.00	0.93 (0.82 1.06)	1.02 (0.83 1.25)
Rectum	Cases	1115		354	501	195	65
	HR (95% CI)*	0.98 (0.94 1.03)	0.42**	1.12 (0.98 1.29)	1.00	0.97 (0.82 1.14)	1.04 (0.80 1.35)
	HR (95% CI)†	0.97 (0.93 1.02)	0.28	**1.20 (1.03 1.39)**	1.00	0.97 (0.82 1.15)	1.03 (0.79 1.35)
	HR (95% CI)^e, g^	0.97 (0.93 1.02)	0.28	1.16 (0.999 1.36)	1.00	0.96 (0.81 1.15)	0.999 (0.76 1.32)
Thyroid	Cases	237		**82**	106	35	14
	HR (95% CI)*	1.02 (0.92 1.12)	0.76	1.31 (0.98 1.76)	1.00	1.10 (0.75 1.61)	1.32 (0.76 2.31)
	HR (95% CI)†	1.01 (0.91 1.11)	0.86	1.36 (0.99 1.87)	1.00	1.08 (0.73 1.59)	1.28 (0.73 2.25)
	HR (95% CI)	1.01 (0.91 1.11)	0.86	1.36 (0.99 1.87)	1.00	1.08 (0.73 1.59)	1.28 (0.73 2.25)
Brain tumours	Cases	463		130	221	87	25
	HR (95% CI)*	1.02 (0.95 1.09)	0.62	0.95 (0.77 1.19)	1.00	1.02 (0.79 1.31)	0.93 (0.61 1.40)
	HR (95% CI)†	1.03 (0.96 1.10)	0.39	0.92 (0.72 1.17)	1.00	1.03 (0.80 1.34)	0.96 (0.63 1.47)
	HR (95% CI)^f^	1.03 (0.96 1.10)	0.38	0.92 (0.72 1.17)	1.00	1.04 (0.80 1.34)	0.97 (0.63 1.48)
Haematological malignancies	Cases	2446		714	1137	445	150
	HR (95% CI)*	1.03 (0.998 1.06)	0.06	0.95 (0.87 1.05)	1.00	1.02 (0.92 1.14)	1.14 (0.96 1.36)
	HR (95% CI)†	1.02 (0.99 1.05)	0.24	0.95 (0.86 1.06)	1.00	0.997 (0.89 1.12)	1.11 (0.93 1.32)
	HR (95% CI)	1.02 (0.99 1.05)	0.24	0.95 (0.86 1.06)	1.00	0.997 (0.89 1.12)	1.11 (0.93 1.32)
Non-Hodgkin’s lymphoma	Cases	1182		349	545	225	63
	HR (95% CI)*	1.02 (0.98 1.06)	0.37	0.97 (0.85 1.11)	1.00	1.09 (0.94 1.28)	1.02 (0.78 1.32)
	HR (95% CI)†	1.01 (0.97 1.06)	0.65	0.996 (0.86 1.15)	1.00	1.07 (0.91 1.26)	1.03 (0.79 1.34)
	HR (95% CI)	1.01 (0.97 1.06)	0.65	0.996 (0.86 1.15)	1.00	1.07 (0.91 1.26)	1.03 (0.79 1.34)

*Models adjusted for age and sex (total observations = 467,656)

†Models adjusted for age, sex, ethnicity (white/other), deprivation index (quintiles), education (University degree, A-levels/HNC/HND/NVQ, GCSE/O-level/CSE, OTHER, None), fruit and vegetable intake (< 5 portions/day, ≥5 portions/day), BMI (kg/m2), height (m), smoking status (never, former light smoker [< 20 pack-years], former heavy smoker [≥20 pack-years], current light smoker [< 20 pack-years], current heavy smoker [≥20 pack-years]) and alcohol intake (never, former, current [<once/week], current [≥once/week])

^a^Additional site-specific covariates in the final model include use of sun/UV protection (Never/rarely/sometimes; most of the time/always; do not go out in sunshine)

^b^Additional site-specific covariates in the final model include HRT use (ever used/never used), oral contraceptive use (ever used/never used), number of live births (0, 1, 2, 3+ live births), age at menarche (early menarche [< 12 years], menarche at 12–14 years, late menarche [≥15 years]), age at menopause (< 40 years, 40–44 years, 45–49 years, 50–54 years, 55–59 years, 60–64 years, ≥65 years, not had menopause/unsure), hysterectomy status (had hysterectomy, not had hysterectomy/unsure)

^c^Additional site-specific covariates in the final model include HRT use (ever used/never used), oral contraceptive use (ever used/never used), number of live births (0, 1, 2, 3+ live births), age at menarche (early menarche [< 12 years], menarche at 12–14 years, late menarche [≥15 years]), age at menopause (< 40 years, 40–44 years, 45–49 years, 50–54 years, 55–59 years, 60–64 years, ≥65 years, not had menopause/unsure), hysterectomy status (had hysterectomy, not had hysterectomy/unsure)

^d^Additional site-specific covariates in the final model include diabetes at baseline (yes/no)

^e^Additional site-specific covariates in the final model include diabetes at baseline (yes/no), aspirin use (regular use/non-regular use or no use), HRT use (ever used/never used; females only), red meat intake (portion/week), processed meat intake (portion/week)

^f^Final model also adjusted for waist-hip ratio (> 94 cm in men, > 80 cm in women)

^f(males)^For cancer sites which were adjusted for different sets of covariates for males and females (colorectal, colon, rectum), this indicates that the final model for male participants was also adjusted for waist-hip ratio (> 94 cm in men)

^g^Results for males and females combined using meta-analysis as covariates are different

^h^Final model also adjusted for family history of cancer (mother/father/sibling had cancer, no family history)

**Schoenfeld test indicated potential violation of the proportional hazards assumption (*p* < 0.05)

Participants who reported > 3 h using computers had a higher risk of lung cancer (HR 1.36, 95% CI: 1.12, 1.65) compared to participants who reported ≤1 h of daily recreational computer time.

### Association of site-specific cancer risk and daily total recreational screen time

Table 6 shows the association between a 1-h increase in daily total recreational screen time and site-specific cancer risk. A 1-h increase in daily total recreational screen time was associated with a higher risk of lung cancer (HR 1.03, 95% CI: 1.004, 1.05).

**Table 6:** Results of Cox proportional hazards analyses investigating the association between self-report daily total screen time and cancer incidence

		1 h increase in total screen time	*p*-value	≤1 h	1- ≤ 4 h (reference)	4- ≤ 8 h	> 8 h
Person-years		3,474,425		254,147	2,111,765	997,699	110,815
Skin, melanoma	Cases	1614		101	960	504	49
	HR (95% CI)*	0.99 (0.97 1.02)	0.64**	0.93 (0.76 1.14)	1.00	1.02 (0.91 1.13)	0.98 (0.73 1.30)
	HR (95% CI)†	1.01 (0.98 1.03)	0.62	0.97 (0.78 1.19)	1.00	1.07 (0.96 1.20)	1.06 (0.78 1.44)
	HR (95% CI)^a^	1.01 (0.98 1.03)	0.70	0.99 (0.80 1.22)	1.00	1.07 (0.95 1.19)	1.07 (0.79 1.45)
Oropharyngeal	Cases	552		24	312	191	25
	HR (95% CI)*	**1.05 (1.01 1.09)**	**0.009**	0.69 (0.46 1.05)	1.00	1.18 (0.99 1.42)	1.39 (0.92 2.09)
	HR (95% CI)†	1.02 (0.98 1.06)	0.41	0.69 (0.45 1.04)	1.00	1.10 (0.91 1.32)	1.09 (0.71 1.67)
	HR (95% CI)	1.02 (0.98 1.06)	0.41	0.69 (0.45 1.04)	1.00	1.10 (0.91 1.32)	1.09 (0.71 1.67)
Lung	Cases	2014		119	995	774	126
	HR (95% CI)*	**1.11 (1.09 1.13)**	< 0.001**	1.13 (0.93 1.37)	1.00	**1.37 (1.25 1.50)**	**2.49 (2.07 3.00)**
	HR (95% CI)†	**1.02 (1.003 1.04)**	0.03**	1.13 (0.92 1.38)	1.00	1.05 (0.95 1.16)	**1.42 (1.16 1.72)**
	HR (95% CI)^h^	**1.03 (1.004 1.05)**	0.02**	1.12 (0.91 1.37)	1.00	1.05 (0.95 1.16)	**1.45 (1.19 1.77)**
Breast (female only)	Cases	5609		418	3526	1522	143
	HR (95% CI)*	1.01 (0.996 1.02)	0.16**	0.91 (0.82 1.004)	1.00	0.97 (0.92 1.04)	1.08 (0.91 1.27)
	HR (95% CI)†	1.003 (0.99 1.02)	0.64**	0.94 (0.85 1.04)	1.00	0.95 (0.90 1.02)	1.08 (0.91 1.29)
	HR (95% CI)^b, h^	1.01 (0.99 1.02)	0.37**	0.93 (0.83 1.04)	1.00	0.97 (0.90 1.04)	1.11 (0.92 1.34)
Uterus	Cases	856		70	504	264	18
	HR (95% CI)*	1.03 (0.99 1.06)	0.14	1.11 (0.87 1.43)	1.00	1.12 (0.96 1.30)	0.99 (0.62 1.58)
	HR (95% CI)†	0.97 (0.93 1.004)	0.08	1.15 (0.88 1.50)	1.00	0.93 (0.80 1.09)	**0.57 (0.33 0.97)**
	HR (95% CI)^c^	0.97 (0.94 1.01)	0.17	1.16 (0.89 1.53)	1.00	0.93 (0.79 1.09)	0.66 (0.38 1.12)
Ovary	Cases	561		44	354	149	14
	HR (95% CI)*	0.99 (0.95 1.04)	0.75	0.99 (0.72 1.35)	1.00	0.90 (0.74 1.09)	1.08 (0.63 1.85)
	HR (95% CI)†	0.997 (0.95 1.04)	0.91	0.98 (0.71 1.35)	1.00	0.90 (0.74 1.11)	1.14 (0.66 1.95)
	HR (95% CI)	0.997 (0.95 1.04)	0.91	0.98 (0.71 1.35)	1.00	0.90 (0.74 1.11)	1.14 (0.66 1.95)
Prostate	Cases	5898		335	3340	2032	191
	HR (95% CI)*	**0.98 (0.97 0.99)**	< 0.001**	1.07 (0.95 1.19)	1.00	0.96 (0.91 1.01)	**0.86 (0.74 0.99)**
	HR (95% CI)†	0.99 (0.98 1.004)	0.16**	1.06 (0.94 1.19)	1.00	1.01 (0.95 1.07)	0.94 (0.81 1.10)
	HR (95% CI)^h^	0.99 (0.98 1.005)	0.21**	1.05 (0.93 1.18)	1.00	1.01 (0.95 1.07)	0.94 (0.81 1.10)
Oesophagus	Cases	528		28	272	216	12
	HR (95% CI)*	**1.05 (1.02 1.10)**	**0.006**	1.02 (0.69 1.50)	1.00	**1.34 (1.12 1.60)**	0.75 (0.42 1.34)
	HR (95% CI)†	1.003 (0.96 1.05)	0.89	1.13 (0.77 1.68)	1.00	1.13 (0.94 1.36)	**0.54 (0.29 0.99)**
	HR (95% CI)^f^	1.001 (0.96 1.04)	0.95	1.15 (0.77 1.69)	1.00	1.12 (0.93 1.36)	**0.54 (0.29 0.99)**
Stomach	Cases	348		14	177	141	16
	HR (95% CI)*	**1.08 (1.04 1.13)**	**< 0.001**	0.77 (0.45 1.33)	1.00	**1.37 (1.09 1.71)**	1.58 (0.95 2.63)
	HR (95% CI)†	1.03 (0.98 1.08)	0.19	0.75 (0.43 1.32)	1.00	1.12 (0.89 1.42)	1.07 (0.61 1.85)
	HR (95% CI)	1.03 (0.98 1.08)	0.19	0.75 (0.43 1.32)	1.00	1.12 (0.89 1.42)	1.07 (0.61 1.85)
Oesophagus and stomach	Cases	870		42	444	356	28
	HR (95% CI)*	**1.07 (1.04 1.10)**	**< 0.001**	0.93 (0.68 1.28)	1.00	**1.36 (1.18 1.57)**	1.09 (0.74 1.59)
	HR (95% CI)†	1.02 (0.98 1.05)	0.31	0.99 (0.72 1.36)	1.00	1.14 (0.98 1.32)	0.76 (0.50 1.14)
	HR (95% CI)^f^	1.02 (0.98 1.05)	0.34	0.99 (0.72 1.37)	1.00	1.13 (0.98 1.31)	0.76 (0.50 1.14)
Hepatobiliary tract	Cases	446		22	249	156	19
	HR (95% CI)*	**1.05 (1.01 1.10)**	**0.02**	0.84 (0.54 1.29)	1.00	1.11 (0.90 1.35)	1.45 (0.91 2.31)
	HR (95% CI)†	1.01 (0.96 1.05)	0.73	0.82 (0.52 1.30)	1.00	0.95 (0.77 1.18)	1.05 (0.65 1.72)
	HR (95% CI)	1.01 (0.96 1.05)	0.73	0.82 (0.52 1.30)	1.00	0.95 (0.77 1.18)	1.05 (0.65 1.72)
Pancreatic	Cases	604		30	333	220	21
	HR (95% CI)*	**1.04 (1.004 1.08)**	**0.03**	0.85 (0.58 1.23)	1.00	**1.17 (0.99 1.39)**	1.22 (0.78 1.89)
	HR (95% CI)†	1.02 (0.98 1.06)	0.37	0.86 (0.58 1.26)	1.00	1.10 (0.92 1.31)	1.01 (0.64 1.60)
	HR (95% CI)^d^	1.02 (0.98 1.06)	0.45	0.86 (0.58 1.26)	1.00	1.08 (0.91 1.30)	0.99 (0.63 1.57)
Kidney	Cases	771		43	417	279	32
	HR (95% CI)*	**1.04 (1.01. 1.08)**	**0.01**	0.98 (0.71 1.34)	1.00	1.19 (1.02 1.38)	1.38 (0.96 1.98)
	HR (95% CI)†	1.01 (0.97 1.04)	0.67**	1.03 (0.74 1.44)	1.00	1.07 (0.91 1.25)	1.14 (0.79 1.66)
	HR (95% CI)	1.01 (0.97 1.04)	0.67**	1.03 (0.74 1.44)	1.00	1.07 (0.91 1.25)	1.14 (0.79 1.66)
Bladder	Cases	662		24	351	259	28
	HR (95% CI)*	**1.06 (1.02 1.09)**	**0.001**	0.69 (0.46 1.04)	1.00	**1.21 (1.03 1.42)**	1.36 (0.93 2.00)
	HR (95% CI)†	1.01 (0.98 1.05)	0.50**	0.76 (0.50 1.16)	1.00	1.10 (0.93 1.30)	0.99 (0.65 1.53)
	HR (95% CI)	1.01 (0.98 1.05)	0.50**	0.76 (0.50 1.16)	1.00	1.10 (0.93 1.30)	0.99 (0.65 1.53)
Colorectal	Cases	3290		180	1896	1096	118
	HR (95% CI)*	**1.02 (1.002 1.04)**	0.03**	0.88 (0.76 1.03)	1.00	1.04 (0.97 1.12)	1.16 (0.97 1.40)
	HR (95% CI)†	1.01 (0.99 1.03)	0.41**	0.92 (0.79 1.08)	1.00	1.003 (0.93 1.08)	1.11 (0.91 1.35)
	HR (95% CI)^e, g, f (males)^	1.01 (0.99 1.02)	0.58**	0.89 (0.76 1.04)	1.00	0.99 (0.91 1.07)	1.08 (0.88 1.32)
Colon	Cases	2110		111	1202	721	76
	HR (95% CI)*	**1.03 (1.01 1.05)**	0.003**	0.86 (0.70 1.04)	1.00	1.08 (0.99 1.19)	1.21 (0.96 1.53)
	HR (95% CI)†	1.02 (0.998 1.04)	0.08**	0.91 (0.75 1.11)	1.00	1.04 (0.95 1.15)	1.16 (0.91 1.48)
	HR (95% CI)^e, g, f (males)^	1.02 (0.99 1.04)	0.15**	0.90 (0.73 1.10)	1.00	1.04 (0.94 1.15)	1.12 (0.88 1.44)
Rectum	Cases	1107		64	645	358	40
	HR (95% CI)*	0.998 (0.97 1.03)	0.89**	0.94 (0.73 1.21)	1.00	0.99 (0.87 1.13)	1.10 (0.80 1.51)
	HR (95% CI)†	0.99 (0.96 1.02)	0.38	0.95 (0.73 1.25)	1.00	0.95 (0.83 1.09)	1.04 (0.74 1.46)
	HR (95% CI)^e, g^	0.98 (0.95 1.02)	0.31	0.91 (0.69 1.20)	1.00	0.92 (0.80 1.06)	1.01 (0.72 1.43)
Brain tumours	Cases	458		28	269	145	16
	HR (95% CI)*	1.01 (0.97 1.06)	0.50	0.96 (0.65 1.42)	1.00	0.99 (0.81 1.22)	1.07 (0.65 1.77)
	HR (95% CI)†	1.02 (0.98 1.07)	0.31	0.98 (0.66 1.46)	1.00	1.001 (0.81 1.24)	1.13 (0.67 1.91)
	HR (95% CI)^f^	1.03 (0.98 1.07)	0.28	0.97 (0.65 1.45)	1.00	1.01 (0.81 1.25)	1.13 (0.67 1.92)
Thyroid	Cases	236		15	154	62	5
	HR (95% CI)*	0.99 (0.93 1.06)	0.86	0.78 (0.46 1.32)	1.00	0.87 (0.65 1.18)	0.71 (0.29 1.72)
	HR (95% CI)†	0.999 (0.93 1.07)	0.97	0.76 (0.44 1.32)	1.00	0.88 (0.64 1.20)	0.70 (0.28 1.72)
	HR (95% CI)	0.999 (0.93 1.07)	0.97	0.76 (0.44 1.32)	1.00	0.88 (0.64 1.20)	0.70 (0.28 1.72)
Haematological malignancies	Cases	2427		142	1396	806	83
	HR (95% CI)*	1.02 (0.998 1.04)	0.09	0.95 (0.80 1.12)	1.00	1.04 (0.96 1.14)	1.12 (0.90 1.40)
	HR (95% CI)†	1.01 (0.99 1.03)	0.43	0.96 (0.80 1.15)	1.00	1.02 (0.93 1.12)	1.07 (0.85 1.34)
	HR (95% CI)	1.01 (0.99 1.03)	0.43	0.96 (0.80 1.15)	1.00	1.02 (0.93 1.12)	1.07 (0.85 1.34)
Non-Hodgkin’s lymphoma	Cases	1174		68	675	392	39
	HR (95% CI)*	1.02 (0.99 1.04)	0.28	0.93 (0.72 1.19)	1.00	1.06 (0.93 1.20)	1.10 (0.80 1.52)
	HR (95% CI)†	1.01 (0.98 1.04)	0.43	0.93 (0.72 1.20)	1.00	1.08 (0.95 1.23)	1.06 (0.75 1.48)
	HR (95% CI)	1.01 (0.98 1.04)	0.43	0.93 (0.72 1.20)	1.00	1.08 (0.95 1.23)	1.06 (0.75 1.48)

*Models adjusted for age and sex (total observations = 464,424)

†Models adjusted for age, sex, ethnicity (white/other), deprivation index (quintiles), education (University degree, A-levels/HNC/HND/NVQ, GCSE/O-level/CSE, OTHER, None), fruit and vegetable intake (< 5 portions/day, ≥5 portions/day), BMI (kg/m2), height (m), smoking status (never, former light smoker [< 20 pack-years], former heavy smoker [≥20 pack-years], current light smoker [< 20 pack-years], current heavy smoker [≥20 pack-years]) and alcohol intake (never, former, current [<once/week], current [≥once/week])

^a^Additional site-specific covariates in the final model include use of sun/UV protection (Never/rarely/sometimes; most of the time/always; do not go out in sunshine)

^b^Additional site-specific covariates in the final model include HRT use (ever used/never used), oral contraceptive use (ever used/never used), number of live births (0, 1, 2, 3+ live births), age at menarche (early menarche [< 12 years], menarche at 12–14 years, late menarche [≥15 years]), age at menopause (< 40 years, 40–44 years, 45–49 years, 50–54 years, 55–59 years, 60–64 years, ≥65 years, not had menopause/unsure), hysterectomy status (had hysterectomy, not had hysterectomy/unsure)

^c^Additional site-specific covariates in the final model include HRT use (ever used/never used), oral contraceptive use (ever used/never used), number of live births (0, 1, 2, 3+ live births), age at menarche (early menarche [< 12 years], menarche at 12–14 years, late menarche [≥15 years]), age at menopause (< 40 years, 40–44 years, 45–49 years, 50–54 years, 55–59 years, 60–64 years, ≥65 years, not had menopause/unsure), hysterectomy status (had hysterectomy, not had hysterectomy/unsure)

^d^Additional site-specific covariates in the final model include diabetes at baseline (yes/no)

^e^Additional site-specific covariates in the final model include diabetes at baseline (yes/no), aspirin use (regular use/non-regular use or no use), HRT use (ever used/never used; females only), red meat intake (portion/week), processed meat intake (portion/week)

^f^Final model also adjusted for waist-hip ratio (> 94 cm in men, > 80 cm in women)

^f(males)^For cancer sites which were adjusted for different sets of covariates for males and females (colorectal, colon, rectum), this indicates that the final model for male participants was also adjusted for waist-hip ratio (> 94 cm in men)

^g^Results for males and females combined using meta-analysis as covariates are different

^h^Final model also adjusted for family history of cancer (mother/father/sibling had cancer, no family history)

**Schoenfeld test indicated potential violation of the proportional hazards assumption (*p* < 0.05)

Participants who reported > 8 h of daily total recreational screen time had a higher risk of lung cancer (HR 1.45, 95% CI: 1.19, 1.77) but a lower risk of oesophagus cancer (HR 0.54, 95% CI: 0.29, 0.99) compared to participants who reported 1- ≤ 4 h of daily total recreational screen time.

## Discussion

### Overview of key findings

This large, prospective cohort study indicates that daily recreational screen time was associated with some site-specific cancers (notably oropharyngeal, oesophagus and stomach, colon, and lung cancer), particularly for TV viewing time, albeit mainly small associations were found. Results for oesophagus and stomach cancers, and colon cancers were robust to the omission of cancers occurring within the first two years of follow-up. However, for many of the other cancer sites the associations were attenuated after eliminating cancers diagnosed within two years, suggesting reverse causation.

The results of our isotemporal substitution models revealed a benefit in terms of reduced risk of several site-specific cancers when replacing 1-h/day of TV viewing with 1-h/day of moderate-intensity physical activity or walking. Results were less consistent for daily recreational computer time and daily total recreational screen time, and were often in the opposite direction to daily TV viewing time. This may suggest that the mechanism of action is more nuanced and complex than the act of being sedentary, but that the specific activity undertaken during sedentary time (i.e. watching TV or using the computer) is an important mechanistic driver. Indeed, Patterson et al., (2018) suggested that sedentary behaviour was not a homogeneous behaviour and found that different sedentary behaviours had different determinants [44]. This will be explored further below.

### Daily TV viewing time and site-specific cancer risk

Television viewing was the most common recreational screen time in this population. Our results showed that a 1-h/day increase in TV viewing time was associated with higher risk of oropharyngeal, stomach, oesophagus and stomach, and colon cancers. Compared with our reference group of 1–3 h/day of TV viewing, reporting less than 1-h/day TV viewing was associated with decreased risk of lung, breast, stomach, and oesophagus and stomach cancers. Thus our analytical approach (setting the reference group to 1–3 h/day as opposed to zero hours/day TV viewing time) enables the exploration of the possible benefits of zero TV viewing time hours for these cancers. There is some evidence in the literature that higher levels of physical activity may reduce lung cancer risk. Mechanistically, this is likely to be due to increased respiratory ventilation, which has the effect of reducing the concentration of carcinogenic agents in the lungs [45]. Previous research also provides evidence for a relationship between higher levels of physical activity and lower risk of incident breast cancer due to decreased sex and metabolic hormone levels, decreased adiposity, reductions in insulin resistance and reduced inflammation [41, 46–49]. It is plausible that similar mechanisms could be applied to the relationship between these cancers and recreational screen time.

Previous research has suggested that individuals who have increased TV viewing time tend to have poor lifestyle behaviours, such as being more likely to smoke, eating a poor diet, doing little, if any, physical activity, and being overweight or obese [7]. Further, Ogden et al. (2013) discussed the concept of ‘mindless eating’, where the distraction of watching the TV led to individuals consuming more calories [50]. A review of the literature on sedentary behaviour and biological pathways by Lynch (2010) supported the hypothesised role of adiposity and metabolic dysfunction as mechanisms operant in the association between sedentary behaviour and cancer [7]. Our findings and other evidence would suggest that recreational sedentary behaviour (including screen time) is much more than an act of not being ‘active’ or being in a stationary position for a prolonged period, but rather a range of sedentary behaviours where the ‘activity’ being undertaken while sedentary is very important. Subsequently, the mechanisms of action for the association between sedentary behaviours and cancer risk are likely to act via a number of complex pathways. For example, TV viewing has been associated with increased risk of being obese or overweight [51], and there is also a strong evidence base associating being overweight or obese to increased cancer risk [7, 52]. However, we adjusted for BMI in our models to try to account for this. Known mechanisms associated with body fatness, such as sex hormones, insulin, and inflammation, may explain part of the association between recreational screen time and cancer risk. The association between prolonged TV viewing time and lower levels of vitamin D has also been hypothesised as a possible mechanistic pathway [7, 11]. However, the association between TV viewing and cancer risk may also be explained by unmeasured confounders, as people who do not watch TV are likely to be different from the broader population in a number of ways.

### Daily recreational computer time and site-specific cancer risk

The mean recreational computer use time was 1.1 h/day, which is almost three times less prevalent as a recreational screen time than daily TV viewing time within this UK population. Paradoxically, our findings showed that a 1-h/day increase in recreational computer use was associated with lower risk of oropharyngeal cancer and the results of the categorical analysis showed that 0 h/day of recreational computer use was associated with higher risk of oropharyngeal and ovary cancers compared with ≤1 h/day. Reporting > 3 h/day of recreational computer use was also associated with increased risk of lung cancer compared with ≤1 h/day. It is difficult to compare the findings for computer use with other literature given the explicit exclusion of ‘using a computer at work’ from our measure. Most of the previous literature is focused on occupational sedentary time which largely encompasses computer use [17].

### Daily total recreational screen time and site-specific cancer risk

The mean daily total recreational screen time was almost 4 h/day, reflecting combined TV viewing and recreational computer use time. The most notable associations were observed for an increased risk of lung cancer in both continuous and categorical analysis. Previous literature has demonstrated that household air pollution exposure from solid fuel is associated with high rates of lung cancer, especially in low- and middle-income countries, such as China [53]. However, this seems an unlikely mechanistic pathway in the UK. It is plausible that indoor sedentary behaviour may be linked to increased residential radon exposure which is known to be associated with an increased risk of lung cancer, particularly in European populations [54]. Results were somewhat mixed for other cancers which may be due to the combined nature of essentially two different behaviours (i.e. TV viewing and recreational computer use).

### Findings in relation with other literature

Our observations are somewhat mixed to those previously reported for oesophago-gastric cancer risk [16] and colon cancer risk [17] in relation to sedentary behaviour. However, it is difficult to draw direct comparisons between these studies and our current analysis, since each of those used the lowest category of screen-time exposure as their reference category. Due to our a priori hypothesis that individuals with less than 1-h/day of screen time may have different characteristics, we chose 1–3 h of screen-time as our reference category. This revealed some novel associations not previously identified, such as protective associations for oesophageal and stomach cancers in individuals with the lowest screen-time exposure.

### Strengths and limitations

This study provides a comprehensive overview of recreational screen time for site-specific cancers. By investigating all cancer sites within the same analytical population, using the same measurement tool for recreational screen time, we hoped to reduce the likelihood of differential measurement error explaining any inconsistencies in the association between screen time and site-specific cancer risk. However, we appreciate that investigating a large number of associations in one analysis may have led to spurious findings. The findings from the partition and isotemporal substitution models are the first, to our knowledge, to model the impact of displacing 1-h/day of TV viewing time with more physically active behaviours for site-specific cancer risk. The UK Biobank has previously been criticised for not being a representative sample for physical activity levels, obesity prevalence and other co-morbidities, indicating a healthy volunteer bias. However, the cohort is representative of the UK population in terms of age, sex, ethnicity and deprivation for the targeted age group [15, 55] and a recently published generalisability study suggests that the results of UK Biobank studies can be generalised to England and Scotland [56]. All models were adjusted for important socio-demographic, health and behavioural variables, including BMI, which is hypothesised to be on the causal pathway between screen time and cancer incidence. Some have argued that this may lead to over-adjustment and therefore underestimation of the strength of the tested associations [15]. Due to the large amount of missing data, the analyses were not adjusted for total calorie consumption or dietary habits other than total fruit and vegetable intake, red and processed meat consumption. Further, we have interpreted our effect modification results with caution owing to the number of cancer sites and number of subgroups which have been investigated.

The analysis uses self-report recreational screen time data, which may be subject to social desirability and recall bias, and the measure has not been investigated for criterion validity [15]. However, the estimates are in line with previous population estimates [57, 58]. Although the UK Biobank cohort does measure sedentary behaviour using accelerometers, we were unable to use this data to examine the association with cancer incidence as the follow-up time was too short (mean follow-up time 1.9 years). The nature of the observational study means that we cannot attribute causal interpretations to our results owing to the potential for residual confounding, particularly for alcohol and tobacco-related cancers. During peer-review, our statistical analysis approach was critiqued for not applying causal inference methodologies. It is our opinion that further biological understanding of the associations shown is required to draw such conclusions. However, we accept that collider bias is possible due to potential bi-directional relationships between screen time, the covariates included in our statistical models, and cancer risk. This would affect the precision of the risk estimates shown. Finally, some associations were attenuated when excluding cancers diagnosed within the first two years of follow-up, suggesting that our results could have been affected by a possible reverse causation bias. An alternative explanation is that the results for some sites became non-significant due to the drop in the number of cancer cases resulting from excluding cancers diagnosed over the 2-year period.

### Future research

Due to the small and inconsistent associations demonstrated in this study, further research is needed to explore the varying and possible trivial associations, which may be due to the large sample size of the UK Biobank cohort. Given the contrasting findings for TV viewing time and recreational computer use time, future research should take a more nuanced approach to exploring recreational screen time. This might help provide a better understanding of the underlying mechanisms of action. The literature to date is dominated by daily and weekly duration of sedentary behaviours. Increasing our knowledge about the role of bouts of sedentary behaviour and the impact of breaks in sedentary behaviour could help us develop more specific time-based recommendations and contribute to the development of much needed cancer prevention strategies. Analysing accelerometer data in large prospective cohorts in future will allow such analyses to be conducted. Accelerometer data has been assessed in UK Biobank during secondary waves of data collection, and so this will be possible given longer follow-up in due course. In addition, the current analysis focussed on site-specific cancer risk, but much remains unknown about the interactive effects of physical activity and recreational screen time on cancer mortality. These areas of research have been highlighted as important evidence gaps in the US 2018 physical activity guidelines [6].

## Conclusions

In summary, our findings show that daily recreational screen time was associated with some site-specific cancers (including oesophagus and stomach, and colon cancers), particularly for daily TV viewing time. Our findings were less consistent for daily recreational computer time and daily total recreational screen time. Substitution models showed that replacing 1-h/day of TV viewing with 1-h/day of moderate-intensity physical activity or walking was associated with lower risk of several site-specific cancers (including oropharyngeal, lung, breast, and colorectal). However, further research from large prospective cohort studies are required to replicate these findings.

## Supplementary information

**Additional file 1.** Supplementary file 1: Subgroup analyses and sensitivity analyses.

Supplementary file 2: Testing linearity assumptions using restricted cubic splines.

## Data Availability

The data that support the findings of this study are available from UK Biobank but restrictions apply to the availability of these data, which were used under license for the current study, and so are not publicly available. Data are however available from the authors upon reasonable request and with permission of UK Biobank.

## Abbreviations

AICR: American Institute for Cancer Research
BMI: Body mass index
CI: Confidence intervals
GCSE: General Certificate of Secondary Education
GDPR: General Data Protection Regulation
GORD: Gastro-Oesophageal Reflux Disease
HNC: Higher National Certificate
HND: Higher National Diploma
HR: Hazard Ratio
HRT: Hormone replacement therapy
ICD: International Classification of Diseases
IPAQ: International Physical Activity Questionnaire
METs: Metabolic equivalents
NCDs: Non-communicable diseases
NHS: National Health Service
NVQ: National Vocational Qualification
RR: Relative Risk
SD: Standard Deviation
TV: Television
UK: United Kingdom
US: United States
UV: Ultraviolet
WCRF: World Cancer Research Fund
