# Supplementary material for: The association between recreational screen time and cancer risk: findings from the UK Biobank, a large prospective cohort study
Source: Int J Behav Nutr Phys Act. 2020 Aug 3;17:97. doi: 10.1186/s12966-020-00997-6 (PMC7398067; doi:10.1186/s12966-020-00997-6)
Supplement: Supplementary file 1 — Additional file 1. Supplementary file 1: Subgroup analyses and sensitivity analyses. [file 12966_2020_997_MOESM1_ESM.docx]

**Supplementary file 1: Subgroup analyses and sensitivity analyses**

**Table 1.1. Results of partition models showing the impact on cancer incidence of a 1-hour increase in total daily TV viewing time, daily moderate activity, daily vigorous activity or daily walking time, holding the other activities constant**

|  |  |  |  |  |  |  |
| --- | --- | --- | --- | --- | --- | --- |
|  |  | 1-hour increase in daily TV viewing time | 1-hour increase in daily moderate activity | 1-hour increase in daily vigorous activity | 1-hour increase in daily walking time |  |
| **Skin melanoma**  [cases=1256] | HR (95% CI)^a^ | 1.01 (0.97, 1.05) | 0.99 (0.90, 1.09) | 0.98 (0.82, 1.18) | 1.03 (0.95, 1.12) |  |
| **Oropharyngeal**  [cases=411] | HR (95% CI) | **1.11 (1.05, 1.17)** | 1.01 (0.86, 1.19) | 0.96 (0.70, 1.31) | 0.87 (0.75, 1.02) |  |
| **Lung**  [cases=1355] | HR (95% CI)^h^ | **1.04 (1.01, 1.07)** | 1.04 (0.96, 1.13) | 0.88 (0.74, 1.04) | 0.93 (0.86, 1.0004) |  |
| **Breast (female only)**  [cases=3454] | HR (95% CI)^b, h^ | 1.005 (0.98, 1.03) | **0.91 (0.86, 0.96)** | 1.02 (0.90, 1.16) | 0.995 (0.95, 1.05) |  |
| **Uterus**  [cases=570] | HR (95% CI)^c^ | 0.98 (0.93, 1.03) | 0.98 (0.85, 1.14) | 1.03 (0.74, 1.42) | 0.97 (0.85, 1.10) |  |
| **Ovary**  [cases=405] | HR (95% CI) | 1.02 (0.95, 1.08) | 1.11 (0.95, 1.29) | 1.14 (0.83, 1.57) | 0.98 (0.85, 1.13) |  |
| **Prostate**  [cases=4629] | HR (95% CI) | 0.99 (0.97, 1.01) | 0.997 (0.95, 1.04) | 1.04 (0.96, 1.13) | 0.99 (0.95, 1.03) |  |
| **Oesophagus**  [cases=386] | HR (95% CI)^f^ | 1.02 (0.96, 1.08) | 1.11 (0.96, 1.29) | 1.08 (0.82, 1.44) | 0.93 (0.80, 1.07) |  |
| **Stomach**  [cases=264] | HR (95% CI) | 1.04 (0.97, 1.12) | 1.10 (0.92, 1.33) | 0.80 (0.54, 1.19) | 0.95 (0.80, 1.14) |  |
| **Oesophagus and stomach**  [cases=644] | HR (95% CI) | 1.03 (0.99, 1.08) | 1.12 (0.99, 1.25) | 0.97 (0.77, 1.22) | 0.93 (0.83, 1.05) |  |
| **Hepatobiliary tract**  [cases=331] | HR (95% CI) | 1.02 (0.96, 1.09) | 0.85 (0.71, 1.03) | 1.03 (0.73, 1.45) | 1.05 (0.89, 1.22) |  |
| **Pancreatic**  [cases=467] | HR (95% CI)^d^ | 1.03 (0.98, 1.09) | 1.11 (0.97, 1.27) | 0.95 (0.71, 1.27) | 0.98 (0.86, 1.12) |  |
| **Kidney**  [cases=559] | HR (95% CI) | 0.997 (0.95, 1.05) | 1.01 (0.88, 1.15) | 1.12 (0.87, 1.43) | 0.95 (0.83, 1.07) |  |
| **Bladder**  [cases=502] | HR (95% CI) | 1.05 (0.99, 1.10) | 1.03 (0.90, 1.18) | 0.86 (0.66, 1.14) | 1.08 (0.95, 1.22) |  |
| **Colorectal**  [cases=2405] | HR (95% CI)^e, g, f (males)^ | 1.02 (0.996, 1.05) | 0.94 (0.88, 1.004) | 1.02 (0.90, 1.16) | 1.03 (0.97, 1.09) |  |
| **Colon**  [cases=1530] | HR (95% CI)^e, g, f (males)^ | 1.03 (0.99, 1.06) | **0.89 (0.81, 0.97)** | 0.99 (0.83, 1.17) | 1.03 (0.95, 1.11) |  |
| **Rectum**  [cases=821] | HR (95% CI)^e, g^ | 1.02 (0.98, 1.07) | 1.02 (0.91, 1.13) | 1.09 (0.89, 1.33) | 1.03 (0.93, 1.13) |  |
| **Brain tumours**  [cases=345] | HR (95% CI) | 1.03 (0.96, 1.11) | 0.87 (0.73, 1.05) | 0.88 (0.61, 1.26) | 1.08 (0.92, 1.26) |  |
| **Thyroid**  [cases=181] | HR (95% CI) | 1.03 (0.93, 1.14) | 0.97 (0.76, 1.25) | 0.83 (0.48, 1.43) | 1.10 (0.90, 1.36) |  |
| **Haematological malignancies**  [cases=1794] | HR (95% CI) | 1.01 (0.97, 1.04) | 0.98 (0.91, 1.06) | 1.08 (0.94, 1.24) | 0.999 (0.93, 1.07) |  |

All models were adjusted for age, sex, ethnicity (white/other), deprivation index (quintiles), education (University degree, A-levels/HNC/HND/NVQ, GCSE/O-level/CSE, OTHER, None), fruit and vegetable intake (<5 portions/day, ≥5 portions/day), BMI (kg/m2), height (m), smoking status (never, former light smoker [<20 pack-years], former heavy smoker [≥20 pack-years], current light smoker [<20 pack-years], current heavy smoker [≥20 pack-years]) and alcohol intake (never, former, current [<once/week], current [≥once/week]).

^a^Additional site-specific covariates in the final model include use of sun/UV protection (Never/rarely/sometimes; most of the time/always; do not go out in sunshine).

^b^Additional site-specific covariates in the final model include HRT use (ever used/never used), oral contraceptive use (ever used/never used), number of live births (0, 1, 2, 3+ live births), age at menarche (early menarche [<12 years], menarche at 12-14 years, late menarche [≥15 years]), age at menopause (<40 years, 40-44 years, 45-49 years, 50-54 years, 55-59 years, 60-64 years, ≥65 years, not had menopause/unsure), hysterectomy status (had hysterectomy, not had hysterectomy/unsure).

^c^Additional site-specific covariates in the final model include HRT use (ever used/never used), oral contraceptive use (ever used/never used), number of live births (0, 1, 2, 3+ live births), age at menarche (early menarche [<12 years], menarche at 12-14 years, late menarche [≥15 years]), age at menopause (<40 years, 40-44 years, 45-49 years, 50-54 years, 55-59 years, 60-64 years, ≥65 years, not had menopause/unsure), hysterectomy status (had hysterectomy, not had hysterectomy/unsure).

^d^Additional site-specific covariates in the final model include diabetes at baseline (yes/no).

^e^Additional site-specific covariates in the final model include diabetes at baseline (yes/no), aspirin use (regular use/non-regular use or no use), HRT use (ever used/never used; females only), red meat intake (portion/week), processed meat intake (portion/week).

^f^Final model also adjusted for waist-hip ratio (>94cm in men, >80cm in women).

^f(males)^For cancer sites which were adjusted for different sets of covariates for males and females (colorectal, colon, rectum), this indicates that the final model for male participants was also adjusted for waist-hip ratio (>94cm in men).

^g^Results for males and females combined using meta-analysis as covariates are different.

^h^Final model also adjusted for family history of cancer (mother/father/sibling had cancer, no family history).

**Table 1.2. Results of Cox proportional hazards analyses investigating the association between a 1-hour increase in self-report daily TV viewing time and cancer incidence for subgroups defined by participant sex**

|  |  |  |  |  |  |
| --- | --- | --- | --- | --- | --- |
|  |  | Females | Males | Wald test for interaction |  |
| Skin, melanoma | Cases | 779 | 795 |  |  |
|  | HR (95% CI)^a^ | 1.02 (0.97 1.07) | 0.99 (0.95 1.04) | p=0.26 |  |
| Oropharyngeal | Cases | 177 | 356 |  |  |
|  | HR (95% CI) | 1.05 (0.96 1.15) | **1.07 (1.01 1.13)** | p=0.46 |  |
| Lung | Cases | 881 | 976 |  |  |
|  | HR (95% CI)^g^ | 0.998 (0.96 1.04) | **1.04 (1.01 1.07)** | p=0.0104 |  |
| Breast (female only) | Cases | 4457 | - |  |  |
|  | HR (95% CI)^b, g^ | 1.01 (0.99 1.03) | - | - |  |
| Uterus | Cases | 748 | - |  |  |
|  | HR (95% CI)^c^ | 0.97 (0.93 1.02) | - | - |  |
| Ovary | Cases | 546 | - |  |  |
|  | HR (95% CI) | 1.02 (0.96 1.08) | - | - |  |
| Prostate | Cases | - | 5565 |  |  |
|  | HR (95% CI)^g^ | - | 0.99 (0.97 1.01) | - |  |
| Oesophagus | Cases | 124 | 378 |  |  |
|  | HR (95% CI)^f^ | 1.03 (0.92 1.15) | 1.02 (0.97 1.08) | p=0.19 |  |
| Stomach | Cases | 97 | 235 |  |  |
|  | HR (95% CI) | 1.10 (0.99 1.23) | 1.05 (0.98 1.12) | p=0.98 |  |
| Oesophagus and stomach | Cases | 221 | 607 |  |  |
|  | HR (95% CI) | 1.06 (0.98 1.15) | 1.04 (0.99 1.09) | p=0.30 |  |
| Hepatobiliary tract | Cases | 179 | 249 |  |  |
|  | HR (95% CI) | 1.05 (0.96 1.15) | 0.99 (0.92 1.07) | p=0.92 |  |
| Pancreatic | Cases | 262 | 316 |  |  |
|  | HR (95% CI)^d^ | 1.01 (0.93 1.09) | 1.06 (0.99 1.12) | p=0.39 |  |
| Kidney | Cases | 261 | 477 |  |  |
|  | HR (95% CI) | **1.08 (1.001 1.16)** | 0.95 (0.90 1.01) | p=0.02 |  |
| Bladder | Cases | 145 | 483 |  |  |
|  | HR (95% CI) | 1.06 (0.96 1.16) | 1.03 (0.98 1.09) | p=0.47 |  |
| Colorectal | Cases | 1325 | 1738 |  |  |
|  | HR (95% CI)^e, f (males)^ | 1.004 (0.97 1.04) | 1.03 (0.997 1.06) | - |  |
| Colon | Cases | 932 | 1040 |  |  |
|  | HR (95% CI)^e, f (males)^ | 1.02 (0.98 1.07) | **1.04 (1.01 1.08)** | - |  |
| Rectum | Cases | 347 | 679 |  |  |
|  | HR (95% CI)^e^ | 0.99 (0.92 1.06) | 0.99 (0.95 1.04) | - |  |
| Brain tumours | Cases | 166 | 268 |  |  |
|  | HR (95% CI) | 1.06 (0.96 1.17) | 1.03 (0.96 1.11) | p=0.94 |  |
| Thyroid | Cases | 168 | 57 |  |  |
|  | HR (95% CI) | 0.98 (0.88 1.09) | 1.06 (0.90 1.24) | p=0.54 |  |
| Haematological malignancies | Cases | 1023 | 1302 |  |  |
|  | HR (95% CI) | 0.98 (0.94 1.03) | 1.01 (0.98 1.05) | p=0.49 |  |
| Non-Hodgkin’s lymphoma | Cases | 526 | 604 |  |  |
|  | HR (95% CI) | 0.99 (0.94 1.05) | 1.03 (0.98 1.08) | p=0.55 |  |

All models were adjusted for age, ethnicity (white/other), deprivation index (quintiles), education (University degree, A-levels/HNC/HND/NVQ, GCSE/O-level/CSE, OTHER, None), fruit and vegetable intake (<5 portions/day, ≥5 portions/day), BMI (kg/m2), height (m), smoking status (never, former light smoker [<20 pack-years], former heavy smoker [≥20 pack-years], current light smoker [<20 pack-years], current heavy smoker [≥20 pack-years]) and alcohol intake (never, former, current [<once/week], current [≥once/week]).

^a^Additional site-specific covariates in the final model include use of sun/UV protection (Never/rarely/sometimes; most of the time/always; do not go out in sunshine).

^b^Additional site-specific covariates in the final model include HRT use (ever used/never used), oral contraceptive use (ever used/never used), number of live births (0, 1, 2, 3+ live births), age at menarche (early menarche [<12 years], menarche at 12-14 years, late menarche [≥15 years]), age at menopause (<40 years, 40-44 years, 45-49 years, 50-54 years, 55-59 years, 60-64 years, ≥65 years, not had menopause/unsure), hysterectomy status (had hysterectomy, not had hysterectomy/unsure).

^c^Additional site-specific covariates in the final model include HRT use (ever used/never used), oral contraceptive use (ever used/never used), number of live births (0, 1, 2, 3+ live births), age at menarche (early menarche [<12 years], menarche at 12-14 years, late menarche [≥15 years]), age at menopause (<40 years, 40-44 years, 45-49 years, 50-54 years, 55-59 years, 60-64 years, ≥65 years, not had menopause/unsure), hysterectomy status (had hysterectomy, not had hysterectomy/unsure).

^d^Additional site-specific covariates in the final model include diabetes at baseline (yes/no).

^e^Additional site-specific covariates in the final model include diabetes at baseline (yes/no), aspirin use (regular use/non-regular use or no use), HRT use (ever used/never used; females only), red meat intake (portion/week), processed meat intake (portion/week).

^f^Final model also adjusted for waist-hip ratio (>94cm in men, >80cm in women).^f(males)^For cancer sites which were adjusted for different sets of covariates for males and females (colorectal, colon, rectum), this indicates that the final model for male participants was also adjusted for waist-hip ratio (>94cm in men).

^g^Final model also adjusted for family history of cancer (mother/father/sibling had cancer, no family history).

**Table 1.3. Results of Cox proportional hazards analyses investigating the association between a 1-hour increase in self-report daily TV viewing time and cancer incidence for subgroups defined by participant age**

|  |  |  |  |  |  |  |  |  |
| --- | --- | --- | --- | --- | --- | --- | --- | --- |
|  |  | <50 years | 50-55 years | 55-60 years | 60-65 years | ≥65 years | Wald test for interaction |  |
| Skin, melanoma | Cases | 236 | 199 | 274 | 475 | 390 |  |  |
|  | HR (95% CI)^a^ | 1.03 (0.94 1.13) | 0.96 (0.86 1.06) | 1.06 (0.98 1.15) | 0.999 (0.94 1.06) | 0.98 (0.92 1.05) | p=0.50 |  |
| Oropharyngeal | Cases | 75 | 91 | 121 | 127 | 119 |  |  |
|  | HR (95% CI) | 1.03 (0.90 1.19) | 1.07 (0.96 1.18) | **1.11 (1.01 1.21)** | **1.11 (1.02 1.22)** | 0.98 (0.88 1.09) | p=0.56 |  |
| Lung | Cases | 66 | 143 | 312 | 623 | 713 |  |  |
|  | HR (95% CI)^h^ | 1.05 (0.93 1.18) | 1.02 (0.94 1.10) | 0.996 (0.94 1.06) | 0.997 (0.95 1.04) | **1.05 (1.01 1.10)** | p=0.75 |  |
| Breast (female only) | Cases | 999 | 622 | 875 | 1201 | 760 |  |  |
|  | HR (95% CI)^b, h^ | 1.01 (0.97 1.06) | 1.004 (0.95 1.06) | 0.96 (0.91 1.01) | 0.99 (0.96 1.03) | **1.06 (1.01 1.11)** | p=0.56 |  |
| Uterus | Cases | 60 | 111 | 171 | 241 | 165 |  |  |
|  | HR (95% CI)^c^ | 0.87 (0.71 1.06) | 0.96 (0.85 1.10) | 0.99 (0.89 1.10) | 0.97 (0.90 1.05) | 0.98 (0.89 1.08) | p=0.15 |  |
| Ovary | Cases | 77 | 64 | 104 | 140 | 161 |  |  |
|  | HR (95% CI) | 1.03 (0.88 1.20) | 1.15 (0.997 1.33) | 1.01 (0.88 1.15) | 1.01 (0.90 1.12) | 0.98 (0.89 1.09) | p=0.52 |  |
| Prostate | Cases | 150 | 379 | 855 | 2106 | 2076 |  |  |
|  | HR (95% CI)^h^ | 0.96 (0.85 1.07) | 0.95 (0.89 1.03) | 0.9997 (0.96 1.04) | 0.98 (0.96 1.01) | 0.99 (0.97 1.02) | p=0.21 |  |
| Oesophagus | Cases | 20 | 38 | 100 | 147 | 197 |  |  |
|  | HR (95% CI)^f^ | 1.15 (0.94 1.42) | 1.12 (0.98 1.27) | 0.97 (0.86 1.09) | 1.07 (0.98 1.18) | 0.97 (0.89 1.06) | p=0.05 |  |
| Stomach | Cases | 28 | 27 | 41 | 106 | 130 |  |  |
|  | HR (95% CI) | **1.17 (1.02 1.34)** | 0.92 (0.71 1.17) | 1.04 (0.88 1.23) | **1.12 (1.01 1.25)** | 1.02 (0.92 1.12) | p=0.29 |  |
| Oesophagus and stomach | Cases | 46 | 65 | 141 | 253 | 323 |  |  |
|  | HR (95% CI) | **1.17 (1.04 1.32)** | 1.06 (0.94 1.20) | 0.99 (0.90 1.09) | **1.09 (1.02 1.17)** | 0.9999 (0.94 1.07) | p=0.13 |  |
| Hepatobiliary tract | Cases | 16 | 47 | 73 | 134 | 158 |  |  |
|  | HR (95% CI) | 0.75 (0.50 1.12) | 0.98 (0.82 1.16) | 1.10 (0.97 1.25) | 0.996 (0.90 1.11) | 1.02 (0.93 1.12) | p=0.06 |  |
| Pancreatic | Cases | 31 | 51 | 104 | 185 | 207 |  |  |
|  | HR (95% CI)^d^ | 0.998 (0.81 1.23) | **1.15 (1.004 1.31)** | 0.95 (0.83 1.08) | 1.02 (0.93 1.11) | 1.06 (0.98 1.15) | p=0.30 |  |
| Kidney | Cases | 56 | 61 | 137 | 246 | 238 |  |  |
|  | HR (95% CI) | 0.95 (0.80 1.13) | 0.89 (0.74 1.07) | 0.99 (0.90 1.10) | 0.97 (0.89 1.05) | 1.06 (0.98 1.14) | p=0.27 |  |
| Bladder | Cases | 21 | 39 | 89 | 211 | 268 |  |  |
|  | HR (95% CI) | 1.02 (0.80 1.28) | 0.996 (0.84 1.18) | 1.02 (0.90 1.15) | 0.998 (0.92 1.09) | **1.08 (1.01 1.16)** | p=0.94 |  |
| Colorectal | Cases | 230 | 340 | 521 | 953 | 1019 |  |  |
|  | HR (95% CI)^e, g, f (males)^ | 0.97 (0.89 1.07) | 1.02 (0.95 1.10) | **1.06 (1.003 1.12)** | 1.02 (0.98 1.06) | 1.01 (0.97 1.05) | p=0.64 (females); p=0.34 (males) |  |
| Colon | Cases | 149 | 196 | 325 | 616 | 686 |  |  |
|  | HR (95% CI)^e, g, f (males)^ | 0.96 (0.85 1.08) | 1.08 (0.99 1.18) | 1.07 (0.999 1.14) | 1.03 (0.98 1.08) | 1.03 (0.99 1.08) | p=0.85 (females); p=0.50 (males) |  |
| Rectum | Cases | 77 | 135 | 183 | 313 | 318 |  |  |
|  | HR (95% CI)^e, g^ | 0.99 (0.85 1.15) | 0.97 (0.86 1.09) | 1.05 (0.95 1.15) | 1.01 (0.94 1.08) | 0.96 (0.89 1.03) | p=0.28 (females); p=0.79 (males) |  |
| Brain tumours | Cases | 45 | 49 | 89 | 133 | 118 |  |  |
|  | HR (95% CI) | 0.92 (0.73 1.16) | 1.06 (0.90 1.25) | 1.04 (0.91 1.19) | 1.03 (0.92 1.15) | 1.07 (0.96 1.19) | p=0.45 |  |
| Thyroid | Cases | 47 | 35 | 45 | 61 | 37 |  |  |
|  | HR (95% CI) | 0.998 (0.83 1.20) | 0.93 (0.73 1.19) | 1.06 (0.88 1.27) | 1.02 (0.86 1.20) | 0.97 (0.78 1.21) | p=0.38 |  |
| Haematological malignancies | Cases | 207 | 231 | 366 | 754 | 767 |  |  |
|  | HR (95% CI) | 1.03 (0.94 1.13) | 1.04 (0.96 1.12) | 0.94 (0.88 1.01) | 0.96 (0.91 1.01) | **1.05 (1.01 1.10)** | p=0.09 |  |
| Non-Hodgkin’s lymphoma | Cases | 95 | 111 | 189 | 353 | 382 |  |  |
|  | HR (95% CI) | 1.03 (0.90 1.18) | 0.97 (0.86 1.09) | 0.92 (0.83 1.02) | 0.99 (0.93 1.06) | **1.08 (1.02 1.14)** | p=0.22 |  |

All models were adjusted for sex, ethnicity (white/other), deprivation index (quintiles), education (University degree, A-levels/HNC/HND/NVQ, GCSE/O-level/CSE, OTHER, None), fruit and vegetable intake (<5 portions/day, ≥5 portions/day), BMI (kg/m2), height (m), smoking status (never, former light smoker [<20 pack-years], former heavy smoker [≥20 pack-years], current light smoker [<20 pack-years], current heavy smoker [≥20 pack-years]) and alcohol intake (never, former, current [<once/week], current [≥once/week]).

^a^Additional site-specific covariates in the final model include use of sun/UV protection (Never/rarely/sometimes; most of the time/always; do not go out in sunshine).

^b^Additional site-specific covariates in the final model include HRT use (ever used/never used), oral contraceptive use (ever used/never used), number of live births (0, 1, 2, 3+ live births), age at menarche (early menarche [<12 years], menarche at 12-14 years, late menarche [≥15 years]), age at menopause (<40 years, 40-44 years, 45-49 years, 50-54 years, 55-59 years, 60-64 years, ≥65 years, not had menopause/unsure), hysterectomy status (had hysterectomy, not had hysterectomy/unsure).

^c^Additional site-specific covariates in the final model include HRT use (ever used/never used), oral contraceptive use (ever used/never used), number of live births (0, 1, 2, 3+ live births), age at menarche (early menarche [<12 years], menarche at 12-14 years, late menarche [≥15 years]), age at menopause (<40 years, 40-44 years, 45-49 years, 50-54 years, 55-59 years, 60-64 years, ≥65 years, not had menopause/unsure), hysterectomy status (had hysterectomy, not had hysterectomy/unsure).

^d^Additional site-specific covariates in the final model include diabetes at baseline (yes/no).

^e^Additional site-specific covariates in the final model include diabetes at baseline (yes/no), aspirin use (regular use/non-regular use or no use), HRT use (ever used/never used; females only), red meat intake (portion/week), processed meat intake (portion/week).

^f^Final model also adjusted for waist-hip ratio (>94cm in men, >80cm in women).

^f(males)^For cancer sites which were adjusted for different sets of covariates for males and females (colorectal, colon, rectum), this indicates that the final model for male participants was also adjusted for waist-hip ratio (>94cm in men).

^g^Results for males and females combined using meta-analysis as covariates are different.

^h^Final model also adjusted for family history of cancer (mother/father/sibling had cancer, no family history).

**Table 1.4. Results of Cox proportional hazards analyses investigating the association between a 1-hour increase in self-report daily TV viewing time and cancer incidence for subgroups defined by deprivation quintile**

|  |  |  |  |  |  |  |  |  |
| --- | --- | --- | --- | --- | --- | --- | --- | --- |
|  |  | 1 (least deprived) | 2 | 3 | 4 | 5 (most deprived) | Wald test for interaction |  |
| Skin, melanoma | Cases | 407 | 378 | 310 | 275 | 204 |  |  |
|  | HR (95% CI)^a^ | 1.06 (0.98 1.13) | 0.98 (0.91 1.05) | 1.03 (0.95 1.11) | 0.95 (0.88 1.03) | 1.005 (0.93 1.09) | p=0.50 |  |
| Oropharyngeal | Cases | 78 | 98 | 105 | 107 | 145 |  |  |
|  | HR (95% CI) | 0.97 (0.82 1.15) | **1.18 (1.05 1.32)** | 0.98 (0.86 1.11) | **1.12 (1.01 1.23)** | 1.05 (0.98 1.12) | p=0.46 |  |
| Lung | Cases | 253 | 291 | 289 | 396 | 628 |  |  |
|  | HR (95% CI)^h^ | **0.87 (0.79 0.95)** | 1.01 (0.93 1.08) | 1.07 (0.998 1.14) | **1.06 (1.004 1.12)** | 1.02 (0.99 1.06) | **p=0.004** |  |
| Breast (female only) | Cases | 963 | 930 | 912 | 930 | 722 |  |  |
|  | HR (95% CI)^b, h^ | **1.06 (1.01 1.11)** | 0.96 (0.92 1.01) | 1.05 (1.0003 1.10) | 0.97 (0.93 1.02) | 0.996 (0.95 1.04) | p=0.03 |  |
| Uterus | Cases | 127 | 159 | 152 | 164 | 146 |  |  |
|  | HR (95% CI)^c^ | 0.99 (0.87 1.12) | 1.04 (0.93 1.16) | **0.88 (0.79 0.99)** | 1.06 (0.96 1.16) | 0.91 (0.83 1.002) | p=0.41 |  |
| Ovary | Cases | 97 | 123 | 120 | 115 | 91 |  |  |
|  | HR (95% CI) | 0.86 (0.74 1.01) | 1.01 (0.89 1.15) | 1.08 (0.96 1.22) | 1.09 (0.98 1.22) | 1.01 (0.89 1.13) | p=0.39 |  |
| Prostate | Cases | 1274 | 1252 | 1176 | 1015 | 849 |  |  |
|  | HR (95% CI)^h^ | 1.01 (0.97 1.05) | 0.98 (0.94 1.02) | 0.98 (0.94 1.02) | 0.98 (0.95 1.02) | 0.98 (0.95 1.02) | p=0.59 |  |
| Oesophagus | Cases | 88 | 91 | 103 | 91 | 129 |  |  |
|  | HR (95% CI)^f^ | 1.01 (0.87 1.17) | 1.07 (0.94 1.22) | 1.11 (0.98 1.25) | 1.07 (0.96 1.20) | 0.95 (0.87 1.04) | p=0.56 |  |
| Stomach | Cases | 51 | 53 | 66 | 81 | 81 |  |  |
|  | HR (95% CI) | 0.95 (0.78 1.17) | 1.07 (0.90 1.26) | 1.14 (0.99 1.30) | 1.10 (0.98 1.23) | 1.03 (0.93 1.14) | p=0.53 |  |
| Oesophagus and stomach | Cases | 138 | 143 | 168 | 171 | 208 |  |  |
|  | HR (95% CI) | 0.99 (0.88 1.12) | 1.07 (0.97 1.19) | **1.13 (1.03 1.23)** | **1.08 (1.001 1.17)** | 0.98 (0.92 1.05) | p=0.36 |  |
| Hepatobiliary tract | Cases | 65 | 78 | 102 | 83 | 100 |  |  |
|  | HR (95% CI) | 1.06 (0.89 1.25) | 0.99 (0.85 1.15) | 0.91 (0.80 1.05) | **1.12 (1.001 1.25)** | 0.99 (0.90 1.09) | p=0.26 |  |
| Pancreatic | Cases | 109 | 117 | 125 | 102 | 125 |  |  |
|  | HR (95% CI)^d^ | 0.92 (0.80 1.06) | 0.99 (0.87 1.12) | 0.99 (0.88 1.11) | 1.04 (0.92 1.17) | **1.12 (1.04 1.20)** | p=0.22 |  |
| Kidney | Cases | 157 | 156 | 176 | 124 | 125 |  |  |
|  | HR (95% CI) | 0.98 (0.88 1.10) | 1.01 (0.91 1.13) | 1.02 (0.93 1.13) | 0.99 (0.89 1.10) | 0.97 (0.89 1.06) | p=0.93 |  |
| Bladder | Cases | 131 | 132 | 131 | 112 | 122 |  |  |
|  | HR (95% CI) | 1.07 (0.95 1.20) | 0.995 (0.89 1.12) | 1.10 (0.99 1.21) | 1.08 (0.97 1.20) | 0.98 (0.90 1.08) | p=0.47 |  |
| Colorectal | Cases | 663 | 649 | 599 | 583 | 569 |  |  |
|  | HR (95% CI)^e, g, f (males)^ | 0.99 (0.94 1.05) | 1.05 (0.99 1.10) | 0.99 (0.93 1.04) | 1.01 (0.96 1.06) | **1.04 (1.000 1.09)** | p=0.52 (females); p=0.65 (males) |  |
| Colon | Cases | 424 | 420 | 388 | 387 | 353 |  |  |
|  | HR (95% CI)^e, g, f (males)^ | 0.99 (0.92 1.06) | **1.09 (1.02 1.16)** | 1.01 (0.94 1.08) | 1.02 (0.96 1.09) | **1.06 (1.01 1.12)** | p=0.51 (females); p=0.55 (males) |  |
| Rectum | Cases | 234 | 221 | 199 | 178 | 194 |  |  |
|  | HR (95% CI)^e, g^ | 1.01 (0.92 1.11) | 0.97 (0.88 1.07) | 0.92 (0.84 1.02) | 1.01 (0.92 1.11) | 1.02 (0.95 1.10) | p=0.29 (females); p=0.69 (males) |  |
| Brain tumours | Cases | 79 | 94 | 92 | 90 | 79 |  |  |
|  | HR (95% CI) | 1.02 (0.87 1.20) | **1.15 (1.02 1.30)** | 0.94 (0.81 1.09) | 1.05 (0.92 1.19) | 1.05 (0.94 1.17) | p=0.32 |  |
| Thyroid | Cases | 49 | 44 | 39 | 53 | 40 |  |  |
|  | HR (95% CI) | 0.99 (0.81 1.22) | 0.92 (0.74 1.15) | 0.95 (0.76 1.20) | 1.03 (0.87 1.23) | 1.05 (0.89 1.24) | p=0.97 |  |
| Haematological malignancies | Cases | 512 | 475 | 469 | 433 | 436 |  |  |
|  | HR (95% CI) | 1.03 (0.97 1.10) | 0.97 (0.91 1.04) | 0.98 (0.92 1.04) | 1.03 (0.98 1.10) | 0.998 (0.95 1.05) | p=0.53 |  |
| Non-Hodgkin’s lymphoma | Cases | 252 | 220 | 224 | 225 | 209 |  |  |
|  | HR (95% CI) | 1.05 (0.96 1.14) | 1.003 (0.91 1.10) | 1.01 (0.93 1.11) | 1.03 (0.95 1.12) | 0.99 (0.92 1.06) | p=0.68 |  |

All models were adjusted for age, sex, ethnicity (white/other), education (University degree, A-levels/HNC/HND/NVQ, GCSE/O-level/CSE, OTHER, None), fruit and vegetable intake (<5 portions/day, ≥5 portions/day), BMI (kg/m2), height (m), smoking status (never, former light smoker [<20 pack-years], former heavy smoker [≥20 pack-years], current light smoker [<20 pack-years], current heavy smoker [≥20 pack-years]) and alcohol intake (never, former, current [<once/week], current [≥once/week]).

^a^Additional site-specific covariates in the final model include use of sun/UV protection (Never/rarely/sometimes; most of the time/always; do not go out in sunshine).

^b^Additional site-specific covariates in the final model include HRT use (ever used/never used), oral contraceptive use (ever used/never used), number of live births (0, 1, 2, 3+ live births), age at menarche (early menarche [<12 years], menarche at 12-14 years, late menarche [≥15 years]), age at menopause (<40 years, 40-44 years, 45-49 years, 50-54 years, 55-59 years, 60-64 years, ≥65 years, not had menopause/unsure), hysterectomy status (had hysterectomy, not had hysterectomy/unsure).

^c^Additional site-specific covariates in the final model include HRT use (ever used/never used), oral contraceptive use (ever used/never used), number of live births (0, 1, 2, 3+ live births), age at menarche (early menarche [<12 years], menarche at 12-14 years, late menarche [≥15 years]), age at menopause (<40 years, 40-44 years, 45-49 years, 50-54 years, 55-59 years, 60-64 years, ≥65 years, not had menopause/unsure), hysterectomy status (had hysterectomy, not had hysterectomy/unsure).

^d^Additional site-specific covariates in the final model include diabetes at baseline (yes/no).

^e^Additional site-specific covariates in the final model include diabetes at baseline (yes/no), aspirin use (regular use/non-regular use or no use), HRT use (ever used/never used; females only), red meat intake (portion/week), processed meat intake (portion/week).

^f^Final model also adjusted for waist-hip ratio (>94cm in men, >80cm in women).

^f(males)^For cancer sites which were adjusted for different sets of covariates for males and females (colorectal, colon, rectum), this indicates that the final model for male participants was also adjusted for waist-hip ratio (>94cm in men).

^g^Results for males and females combined using meta-analysis as covariates are different.

^h^Final model also adjusted for family history of cancer (mother/father/sibling had cancer, no family history).

**Table 1.5. Results of Cox proportional hazards analyses investigating the association between a 1-hour increase in self-report daily TV viewing time and cancer incidence for subgroups defined by smoking status**

|  |  |  |  |  |  |  |  |  |
| --- | --- | --- | --- | --- | --- | --- | --- | --- |
|  |  | Never | Former light smoker | Former heavy smoker | Current light smoker | Current heavy smoker | Wald test for interaction |  |
| Skin, melanoma | Cases | 900 | 448 | 144 | 47 | 35 |  |  |
|  | HR (95% CI)^a^ | 1.001 (0.95 1.05) | 0.998 (0.93 1.07) | 1.03 (0.94 1.13) | 1.13 (0.97 1.30) | 0.86 (0.70 1.06) | p=0.13 |  |
| Oropharyngeal | Cases | 179 | 135 | 70 | 49 | 100 |  |  |
|  | HR (95% CI) | 0.997 (0.90 1.10) | 1.08 (0.97 1.21) | 1.09 (0.97 1.23) | 1.10 (0.98 1.23) | 1.04 (0.96 1.13) | p=0.72 |  |
| Lung | Cases | 256 | 294 | 553 | 178 | 576 |  |  |
|  | HR (95% CI)^h^ | 1.03 (0.95 1.12) | 1.01 (0.94 1.09) | 1.02 (0.97 1.07) | **1.08 (1.01 1.16)** | 1.01 (0.97 1.05) | p=0.04 |  |
| Breast (female only) | Cases | 2604 | 1153 | 302 | 217 | 181 |  |  |
|  | HR (95% CI)^b, h^ | 1.01 (0.98 1.04) | 1.01 (0.97 1.05) | 1.04 (0.97 1.12) | 0.96 (0.88 1.05) | 0.97 (0.89 1.05) | p=0.49 |  |
| Uterus | Cases | 495 | 170 | 51 | 17 | 15 |  |  |
|  | HR (95% CI)^c^ | 1.004 (0.95 1.06) | 0.90 (0.81 1.01) | 0.88 (0.74 1.04) | 1.08 (0.81 1.44) | 0.89 (0.66 1.21) | p=0.36 |  |
| Ovary | Cases | 316 | 138 | 49 | 24 | 19 |  |  |
|  | HR (95% CI) | 1.002 (0.93 1.08) | 1.06 (0.94 1.18) | 0.88 (0.73 1.05) | **1.23 (1.08 1.42)** | 0.96 (0.75 1.23) | p=0.09 |  |
| Prostate | Cases | 2659 | 1697 | 710 | 278 | 222 |  |  |
|  | HR (95% CI)^h^ | 0.99 (0.96 1.01) | 0.99 (0.96 1.03) | 1.01 (0.97 1.06) | 1.01 (0.95 1.09) | **0.88 (0.82 0.95)** | p=0.07 |  |
| Oesophagus | Cases | 154 | 139 | 112 | 38 | 59 |  |  |
|  | HR (95% CI)^f^ | 1.04 (0.94 1.15) | 1.02 (0.92 1.14) | 1.01 (0.91 1.12) | 0.93 (0.77 1.13) | 1.07 (0.95 1.19) | p=0.79 |  |
| Stomach | Cases | 123 | 78 | 72 | 23 | 36 |  |  |
|  | HR (95% CI) | **1.19 (1.09 1.30)** | 1.04 (0.91 1.19) | 1.01 (0.89 1.15) | 0.96 (0.76 1.22) | 0.97 (0.83 1.14) | p=0.34 |  |
| Oesophagus and stomach | Cases | 276 | 216 | 184 | 59 | 93 |  |  |
|  | HR (95% CI) | **1.11 (1.04 1.19)** | 1.03 (0.95 1.12) | 1.01 (0.93 1.10) | 0.95 (0.82 1.11) | 1.04 (0.95 1.14) | p=0.27 |  |
| Hepatobiliary tract | Cases | 164 | 128 | 78 | 25 | 33 |  |  |
|  | HR (95% CI) | 1.03 (0.93 1.14) | 0.99 (0.88 1.11) | 0.95 (0.83 1.08) | 1.13 (0.97 1.31) | 1.03 (0.89 1.20) | p=0.47 |  |
| Pancreatic | Cases | 262 | 139 | 80 | 37 | 60 |  |  |
|  | HR (95% CI)^d^ | 0.99 (0.92 1.08) | 1.07 (0.96 1.19) | **1.12 (1.01 1.25)** | 1.08 (0.92 1.28) | 0.96 (0.85 1.10) | p=0.41 |  |
| Kidney | Cases | 315 | 206 | 115 | 42 | 60 |  |  |
|  | HR (95% CI) | 1.01 (0.94 1.08) | 0.98 (0.89 1.07) | 0.97 (0.87 1.08) | 1.05 (0.89 1.23) | 1.003 (0.88 1.14) | p=0.86 |  |
| Bladder | Cases | 187 | 182 | 141 | 50 | 68 |  |  |
|  | HR (95% CI) | 1.07 (0.98 1.18) | 1.08 (0.99 1.18) | 1.01 (0.92 1.12) | 0.99 (0.84 1.17) | 0.99 (0.88 1.11) | p=0.39 |  |
| Colorectal | Cases | 1407 | 864 | 480 | 155 | 157 |  |  |
|  | HR (95% CI)^e, g, f (males)^ | 1.02 (0.98 1.06) | 1.03 (0.99 1.08) | 0.97 (0.92 1.03) | 1.04 (0.95 1.14) | 1.06 (0.99 1.14) | p=0.61 (females); p=0.36 (males) |  |
| Colon | Cases | 939 | 550 | 295 | 100 | 88 |  |  |
|  | HR (95% CI)^e, g, f (males)^ | 1.03 (0.99 1.08) | 1.05 (0.996 1.11) | 0.97 (0.90 1.04) | 1.10 (0.99 1.21) | **1.10 (1.01 1.21)** | p=0.13 (females); p=0.15 (males) |  |
| Rectum | Cases | 441 | 301 | 178 | 51 | 55 |  |  |
|  | HR (95% CI)^e, g^ | 1.01 (0.94 1.07) | 0.996 (0.92 1.08) | 0.98 (0.89 1.07) | 0.91 (0.77 1.09) | 1.003 (0.88 1.14) | p=0.88 (females); p=0.85 (males) |  |
| Brain tumours | Cases | 212 | 141 | 38 | 20 | 23 |  |  |
|  | HR (95% CI) | 0.996 (0.91 1.09) | 1.04 (0.93 1.16) | **1.18 (1.02 1.36)** | 1.13 (0.94 1.35) | 0.97 (0.80 1.19) | p=0.11 |  |
| Thyroid | Cases | 130 | 57 | 20 | 11 | 7 |  |  |
|  | HR (95% CI) | 1.02 (0.90 1.14) | 0.91 (0.75 1.10) | 1.06 (0.82 1.38) | 1.04 (0.77 1.41) | 1.04 (0.72 1.53) | p=0.94 |  |
| Haematological malignancies | Cases | 1158 | 625 | 298 | 109 | 135 |  |  |
|  | HR (95% CI) | 1.01 (0.97 1.05) | 1.03 (0.97 1.08) | 0.97 (0.91 1.04) | 0.97 (0.86 1.09) | 0.98 (0.91 1.07) | p=0.63 |  |
| Non-Hodgkin’s lymphoma | Cases | 584 | 290 | 134 | 52 | 70 |  |  |
|  | HR (95% CI) | 0.99 (0.94 1.05) | **1.12 (1.04 1.20)** | 0.99 (0.89 1.09) | 0.98 (0.83 1.15) | 0.91 (0.81 1.03) | p=0.15 |  |

All models were adjusted for age, sex, ethnicity (white/other), deprivation index (quintiles), education (University degree, A-levels/HNC/HND/NVQ, GCSE/O-level/CSE, OTHER, None), fruit and vegetable intake (<5 portions/day, ≥5 portions/day), BMI (kg/m2), height (m) and alcohol intake (never, former, current [<once/week], current [≥once/week]).

^a^Additional site-specific covariates in the final model include use of sun/UV protection (Never/rarely/sometimes; most of the time/always; do not go out in sunshine).

^b^Additional site-specific covariates in the final model include HRT use (ever used/never used), oral contraceptive use (ever used/never used), number of live births (0, 1, 2, 3+ live births), age at menarche (early menarche [<12 years], menarche at 12-14 years, late menarche [≥15 years]), age at menopause (<40 years, 40-44 years, 45-49 years, 50-54 years, 55-59 years, 60-64 years, ≥65 years, not had menopause/unsure), hysterectomy status (had hysterectomy, not had hysterectomy/unsure).

^c^Additional site-specific covariates in the final model include HRT use (ever used/never used), oral contraceptive use (ever used/never used), number of live births (0, 1, 2, 3+ live births), age at menarche (early menarche [<12 years], menarche at 12-14 years, late menarche [≥15 years]), age at menopause (<40 years, 40-44 years, 45-49 years, 50-54 years, 55-59 years, 60-64 years, ≥65 years, not had menopause/unsure), hysterectomy status (had hysterectomy, not had hysterectomy/unsure).

^d^Additional site-specific covariates in the final model include diabetes at baseline (yes/no).

^e^Additional site-specific covariates in the final model include diabetes at baseline (yes/no), aspirin use (regular use/non-regular use or no use), HRT use (ever used/never used; females only), red meat intake (portion/week), processed meat intake (portion/week).

^f^Final model also adjusted for waist-hip ratio (>94cm in men, >80cm in women).

^f(males)^For cancer sites which were adjusted for different sets of covariates for males and females (colorectal, colon, rectum), this indicates that the final model for male participants was also adjusted for waist-hip ratio (>94cm in men).

^g^Results for males and females combined using meta-analysis as covariates are different.

^h^Final model also adjusted for family history of cancer (mother/father/sibling had cancer, no family history).

**Table 1.6. Results of Cox proportional hazards analyses investigating the association between a 1-hour increase in self-report daily TV viewing time and cancer incidence for subgroups defined by participant BMI**

|  |  |  |  |  |  |
| --- | --- | --- | --- | --- | --- |
|  |  | Non-obese | Obese | Wald test for interaction |  |
| Skin, melanoma | Cases | 1211 | 363 |  |  |
|  | HR (95% CI)^a^ | 0.99 (0.95 1.03) | 1.04 (0.98 1.11) | p=0.89 |  |
| Oropharyngeal | Cases | 391 | 142 |  |  |
|  | HR (95% CI) | **1.09 (1.04 1.15)** | 0.97 (0.88 1.07) | p=0.96 |  |
| Lung | Cases | 1377 | 480 |  |  |
|  | HR (95% CI)^h^ | 1.03 (0.997 1.06) | 1.01 (0.96 1.06) | p=0.41 |  |
| Breast (female only) | Cases | 3365 | 1092 |  |  |
|  | HR (95% CI)^b, h^ | 1.02 (0.99 1.04) | 0.97 (0.94 1.01) | p=0.02 |  |
| Uterus | Cases | 406 | 342 |  |  |
|  | HR (95% CI)^c^ | 1.02 (0.95 1.09) | 0.94 (0.88 1.00003) | p=0.25 |  |
| Ovary | Cases | 407 | 139 |  |  |
|  | HR (95% CI) | 1.04 (0.97 1.11) | 0.96 (0.86 1.07) | p=0.50 |  |
| Prostate | Cases | 4276 | 1290 |  |  |
|  | HR (95% CI)^h^ | 0.99 (0.97 1.01) | 0.99 (0.96 1.03) | p=0.18 |  |
| Oesophagus | Cases | 341 | 161 |  |  |
|  | HR (95% CI)^f^ | 1.01 (0.95 1.08) | 1.04 (0.96 1.13) | p=0.95 |  |
| Stomach | Cases | 229 | 103 |  |  |
|  | HR (95% CI) | **1.12 (1.05 1.20)** | 0.95 (0.86 1.06) | p=0.03 |  |
| Oesophagus and stomach | Cases | 566 | 262 |  |  |
|  | HR (95% CI) | **1.06 (1.01 1.12)** | 1.01 (0.94 1.07) | p=0.12 |  |
| Hepatobiliary tract | Cases | 262 | 166 |  |  |
|  | HR (95% CI) | 1.02 (0.94 1.10) | 1.003 (0.92 1.09) | p=0.23 |  |
| Pancreatic | Cases | 401 | 177 |  |  |
|  | HR (95% CI)^d^ | 1.04 (0.98 1.11) | 1.02 (0.93 1.10) | p=0.42 |  |
| Kidney | Cases | 465 | 273 |  |  |
|  | HR (95% CI) | 1.004 (0.95 1.07) | 0.98 (0.92 1.06) | p=0.72 |  |
| Bladder | Cases | 424 | 204 |  |  |
|  | HR (95% CI) | 1.03 (0.97 1.09) | 1.05 (0.97 1.13) | p=0.09 |  |
| Colorectal | Cases | 2228 | 835 |  |  |
|  | HR (95% CI)^e, g, f (males)^ | 1.01 (0.99 1.04) | 1.03 (0.99 1.07) | p=0.55 (females); p=0.73 (males) |  |
| Colon | Cases | 1400 | 572 |  |  |
|  | HR (95% CI)^e, g, f (males)^ | 1.03 (0.996 1.07) | 1.04 (0.99 1.09) | p=0.85 (females); p=0.53 (males) |  |
| Rectum | Cases | 770 | 256 |  |  |
|  | HR (95% CI)^e, g^ | 0.98 (0.94 1.03) | 1.01 (0.94 1.09) | p=0.12 (females); p=0.79 (males) |  |
| Brain tumours | Cases | 324 | 110 |  |  |
|  | HR (95% CI) | 1.05 (0.98 1.13) | 1.03 (0.92 1.14) | p=0.64 |  |
| Thyroid | Cases | 157 | 68 |  |  |
|  | HR (95% CI) | 1.03 (0.93 1.15) | 0.95 (0.81 1.11) | p=0.37 |  |
| Haematological malignancies | Cases | 1702 | 623 |  |  |
|  | HR (95% CI) | 1.003 (0.97 1.04) | 0.997 (0.95 1.04) | p=0.31 |  |
| Non-Hodgkin’s lymphoma | Cases | 841 | 289 |  |  |
|  | HR (95% CI) | 1.02 (0.97 1.06) | 1.004 (0.94 1.07) | p=0.20 |  |

All models were adjusted for age, sex, ethnicity (white/other), deprivation index (quintiles), education (University degree, A-levels/HNC/HND/NVQ, GCSE/O-level/CSE, OTHER, None), fruit and vegetable intake (<5 portions/day, ≥5 portions/day), height (m), smoking status (never, former light smoker [<20 pack-years], former heavy smoker [≥20 pack-years], current light smoker [<20 pack-years], current heavy smoker [≥20 pack-years]) and alcohol intake (never, former, current [<once/week], current [≥once/week]).

^a^Additional site-specific covariates in the final model include use of sun/UV protection (Never/rarely/sometimes; most of the time/always; do not go out in sunshine).

^b^Additional site-specific covariates in the final model include HRT use (ever used/never used), oral contraceptive use (ever used/never used), number of live births (0, 1, 2, 3+ live births), age at menarche (early menarche [<12 years], menarche at 12-14 years, late menarche [≥15 years]), age at menopause (<40 years, 40-44 years, 45-49 years, 50-54 years, 55-59 years, 60-64 years, ≥65 years, not had menopause/unsure), hysterectomy status (had hysterectomy, not had hysterectomy/unsure).

^c^Additional site-specific covariates in the final model include HRT use (ever used/never used), oral contraceptive use (ever used/never used), number of live births (0, 1, 2, 3+ live births), age at menarche (early menarche [<12 years], menarche at 12-14 years, late menarche [≥15 years]), age at menopause (<40 years, 40-44 years, 45-49 years, 50-54 years, 55-59 years, 60-64 years, ≥65 years, not had menopause/unsure), hysterectomy status (had hysterectomy, not had hysterectomy/unsure).

^d^Additional site-specific covariates in the final model include diabetes at baseline (yes/no).

^e^Additional site-specific covariates in the final model include diabetes at baseline (yes/no), aspirin use (regular use/non-regular use or no use), HRT use (ever used/never used; females only), red meat intake (portion/week), processed meat intake (portion/week).

^f^Final model also adjusted for waist-hip ratio (>94cm in men, >80cm in women).

^f(males)^For cancer sites which were adjusted for different sets of covariates for males and females (colorectal, colon, rectum), this indicates that the final model for male participants was also adjusted for waist-hip ratio (>94cm in men).

^g^Results for males and females combined using meta-analysis as covariates are different.

^h^Final model also adjusted for family history of cancer (mother/father/sibling had cancer, no family history).

**Table 1.7. Results of Cox proportional hazards analyses investigating the association between a 1-hour increase in self-report daily TV viewing time and cancer incidence for subgroups defined by participant IPAQ physical activity category**

|  |  |  |  |  |  |  |
| --- | --- | --- | --- | --- | --- | --- |
|  |  | Low | Moderate | High | Wald test for interaction |  |
| Skin, melanoma | Cases | 200 | 540 | 516 |  |  |
|  | HR (95% CI)^a^ | 0.995 (0.91, 1.08) | 1.04 (0.98, 1.10) | 0.97 (0.91, 1.04) | p=0.52 |  |
| Oropharyngeal | Cases | 90 | 151 | 170 |  |  |
|  | HR (95% CI) | **1.09 (1.0004, 1.20)** | **1.12 (1.03, 1.22)** | **1.12 (1.02, 1.23)** | p=0.52 |  |
| Lung | Cases | 284 | 555 | 516 |  |  |
|  | HR (95% CI)^h^ | 1.03 (0.97, 1.08) | 1.05 (0.998, 1.10) | 1.03 (0.98, 1.09) | p=0.11 |  |
| Breast (female only) | Cases | 625 | 1570 | 1259 |  |  |
|  | HR (95% CI)^b, h^ | 0.99 (0.94, 1.04) | 0.998 (0.96, 1.03) | 1.02 (0.97, 1.06) | p=0.11 |  |
| Uterus | Cases | 115 | 258 | 197 |  |  |
|  | HR (95% CI)^c^ | 0.92 (0.82, 1.03) | 1.003 (0.92, 1.09) | 0.98 (0.89, 1.09) | p=0.90 |  |
| Ovary | Cases | 64 | 171 | 170 |  |  |
|  | HR (95% CI) | 1.004 (0.86, 1.17) | 1.05 (0.95, 1.16) | 0.97 (0.87, 1.08) | p=0.64 |  |
| Prostate | Cases | 741 | 1890 | 1998 |  |  |
|  | HR (95% CI)^h^ | 1.01 (0.97, 1.06) | 0.98 (0.95, 1.01) | 0.99 (0.96, 1.02) | p=0.87 |  |
| Oesophagus | Cases | 76 | 142 | 168 |  |  |
|  | HR (95% CI)^f^ | 1.03 (0.92, 1.15) | 1.01 (0.92, 1.12) | 1.02 (0.93, 1.13) | p=0.26 |  |
| Stomach | Cases | 53 | 108 | 104 |  |  |
|  | HR (95% CI) | 1.09 (0.98, 1.23) | 0.99 (0.88, 1.11) | 1.11 (0.99, 1.26) | p=0.48 |  |
| Oesophagus and stomach | Cases | 127 | 247 | 271 |  |  |
|  | HR (95% CI) | 1.06 (0.98, 1.15) | 1.01 (0.93, 1.09) | 1.06 (0.98, 1.15) | p=0.27 |  |
| Hepatobiliary tract | Cases | 74 | 138 | 119 |  |  |
|  | HR (95% CI) | 0.97 (0.86, 1.10) | 1.01 (0.91, 1.12) | 1.06 (0.94, 1.19) | p=0.38 |  |
| Pancreatic | Cases | 90 | 190 | 187 |  |  |
|  | HR (95% CI)^d^ | 0.99 (0.89, 1.11) | 1.08 (0.996, 1.18) | 1.001 (0.91, 1.10) | p=0.999 |  |
| Kidney | Cases | 104 | 248 | 207 |  |  |
|  | HR (95% CI) | 0.89 (0.79, 1.01) | 1.01 (0.93, 1.10) | 1.04 (0.95, 1.14) | p=0.29 |  |
| Bladder | Cases | 82 | 207 | 213 |  |  |
|  | HR (95% CI) | 1.08 (0.98, 1.20) | 1.05 (0.97, 1.14) | 1.01 (0.93, 1.11) | p=0.82 |  |
| Colorectal | Cases | 429 | 1008 | 969 |  |  |
|  | HR (95% CI)^e, g, f (males)^ | 0.98 (0.93, 1.04) | **1.04 (1.004, 1.09)** | 1.03 (0.99, 1.08) | p=0.59 (females); p=0.93 (males) |  |
| Colon | Cases | 290 | 658 | 583 |  |  |
|  | HR (95% CI)^e, g, f (males)^ | 1.01 (0.95, 1.08) | **1.06 (1.01, 1.11)** | 1.01 (0.95, 1.07) | p=0.35 (females); p=0.08 (males) |  |
| Rectum | Cases | 131 | 327 | 363 |  |  |
|  | HR (95% CI)^e, g^ | 0.96 (0.86, 1.06) | 1.002 (0.93, 1.08) | **1.09 (1.01, 1.17)** | p=0.49 (females); p=0.004 (males) |  |
| Brain tumours | Cases | 63 | 145 | 137 |  |  |
|  | HR (95% CI) | **1.14 (1.03, 1.26)** | 0.94 (0.84, 1.06) | 1.01 (0.90, 1.14) | p=0.05 |  |
| Thyroid | Cases | 34 | 73 | 74 |  |  |
|  | HR (95% CI) | 1.05 (0.87, 1.26) | 0.92 (0.78, 1.10) | 1.12 (0.97, 1.29) | p=0.34 |  |
| Haematological malignancies | Cases | 320 | 745 | 729 |  |  |
|  | HR (95% CI) | 0.94 (0.88, 1.01) | 0.99 (0.94, 1.04) | 1.05 (0.997, 1.10) | p=0.77 |  |
| Non-Hodgkin’s lymphoma | Cases | 152 | 350 | 362 |  |  |
|  | HR (95% CI) | 0.97 (0.89, 1.07) | 1.02 (0.95, 1.09) | 1.05 (0.98, 1.12) | p=0.54 |  |

All models were adjusted for age, sex, ethnicity (white/other), deprivation index (quintiles), education (University degree, A-levels/HNC/HND/NVQ, GCSE/O-level/CSE, OTHER, None), fruit and vegetable intake (<5 portions/day, ≥5 portions/day), height (m), smoking status (never, former light smoker [<20 pack-years], former heavy smoker [≥20 pack-years], current light smoker [<20 pack-years], current heavy smoker [≥20 pack-years]) and alcohol intake (never, former, current [<once/week], current [≥once/week]).

^a^Additional site-specific covariates in the final model include use of sun/UV protection (Never/rarely/sometimes; most of the time/always; do not go out in sunshine).

^b^Additional site-specific covariates in the final model include HRT use (ever used/never used), oral contraceptive use (ever used/never used), number of live births (0, 1, 2, 3+ live births), age at menarche (early menarche [<12 years], menarche at 12-14 years, late menarche [≥15 years]), age at menopause (<40 years, 40-44 years, 45-49 years, 50-54 years, 55-59 years, 60-64 years, ≥65 years, not had menopause/unsure), hysterectomy status (had hysterectomy, not had hysterectomy/unsure).

^c^Additional site-specific covariates in the final model include HRT use (ever used/never used), oral contraceptive use (ever used/never used), number of live births (0, 1, 2, 3+ live births), age at menarche (early menarche [<12 years], menarche at 12-14 years, late menarche [≥15 years]), age at menopause (<40 years, 40-44 years, 45-49 years, 50-54 years, 55-59 years, 60-64 years, ≥65 years, not had menopause/unsure), hysterectomy status (had hysterectomy, not had hysterectomy/unsure).

^d^Additional site-specific covariates in the final model include diabetes at baseline (yes/no).

^e^Additional site-specific covariates in the final model include diabetes at baseline (yes/no), aspirin use (regular use/non-regular use or no use), HRT use (ever used/never used; females only), red meat intake (portion/week), processed meat intake (portion/week).

^f^Final model also adjusted for waist-hip ratio (>94cm in men, >80cm in women).

^f(males)^For cancer sites which were adjusted for different sets of covariates for males and females (colorectal, colon, rectum), this indicates that the final model for male participants was also adjusted for waist-hip ratio (>94cm in men).

^g^Results for males and females combined using meta-analysis as covariates are different.

^h^Final model also adjusted for family history of cancer (mother/father/sibling had cancer, no family history).

**Table 1.8. Results of Cox proportional hazards analyses investigating the association between a 1-hour increase in self-report daily TV viewing time and cancer incidence for subgroups defined by body fat percentage and physical activity level**

|  |  |  |  |  |  |  |  |
| --- | --- | --- | --- | --- | --- | --- | --- |
|  |  | High/moderate PA and low/optimal BFP | Low PA and low/optimal BFP | High/moderate PA and high BFP | Low PA and high BFP | Wald test for interaction |  |
| Skin, melanoma | Cases | 277 | 323 | 211 | 432 |  |  |
|  | HR (95% CI)^a^ | 0.96 (0.88 1.06) | 1.02 (0.94 1.10) | 1.03 (0.94 1.13) | 1.01 (0.95 1.07) | p=0.11 |  |
| Oropharyngeal | Cases | 53 | 117 | 46 | 189 |  |  |
|  | HR (95% CI) | 1.04 (0.84 1.27) | 1.08 (0.98 1.19) | 1.11 (0.94 1.32) | **1.12 (1.04 1.20)** | p=0.20 |  |
| Lung | Cases | 244 | 276 | 220 | 583 |  |  |
|  | HR (95% CI)^h^ | 1.05 (0.97 1.14) | 1.03 (0.97 1.09) | 1.02 (0.94 1.10) | 1.03 (0.99 1.07) | **p=0.003** |  |
| Breast (female only) | Cases | 1325 | 274 | 1366 | 445 |  |  |
|  | HR (95% CI)^b, h^ | 1.02 (0.98 1.07) | 1.02 (0.94 1.11) | 0.99 (0.95 1.02) | 1.003 (0.95 1.06) | p=0.013 |  |
| Uterus | Cases | 159 | 26 | 273 | 106 |  |  |
|  | HR (95% CI)^c^ | 1.10 (0.98 1.23) | 0.91 (0.68 1.21) | 0.95 (0.87 1.03) | 0.92 (0.82 1.04) | p=0.87 |  |
| Ovary | Cases | 157 | 31 | 170 | 42 |  |  |
|  | HR (95% CI) | 0.99 (0.88 1.11) | 1.08 (0.87 1.36) | 1.03 (0.93 1.14) | 0.9993 (0.83 1.20) | p=0.19 |  |
| Prostate | Cases | - | 1989 | - | 2563 |  |  |
|  | HR (95% CI)^h^ | - | 0.99 (0.96 1.02) | - | 0.99 (0.96 1.01) | p=0.73 |  |
| Oesophagus | Cases | 48 | 78 | 29 | 218 |  |  |
|  | HR (95% CI)^f^ | 0.85 (0.69 1.04) | 1.11 (0.98 1.25) | 1.14 (0.93 1.40) | 1.02 (0.95 1.10) | p=0.13 |  |
| Stomach | Cases | 38 | 61 | 23 | 140 |  |  |
|  | HR (95% CI) | 1.06 (0.84 1.32) | 1.09 (0.95 1.25) | **1.27 (1.05 1.53)** | 1.04 (0.95 1.14) | p=0.35 |  |
| Oesophagus and stomach | Cases | 86 | 137 | 52 | 354 |  |  |
|  | HR (95% CI) | 0.93 (0.80 1.09) | **1.11 (1.01 1.21)** | **1.20 (1.04 1.38)** | 1.04 (0.98 1.10) | p=0.03 |  |
| Hepatobiliary tract | Cases | 43 | 54 | 61 | 165 |  |  |
|  | HR (95% CI) | 1.16 (0.95 1.42) | 0.94 (0.78 1.12) | 0.98 (0.83 1.17) | 1.01 (0.93 1.10) | p=0.13 |  |
| Pancreatic | Cases | 71 | 105 | 76 | 204 |  |  |
|  | HR (95% CI)^d^ | 0.91 (0.77 1.08) | **1.14 (1.03 1.26)** | 0.91 (0.78 1.06) | 1.04 (0.96 1.13) | p=0.03 |  |
| Kidney | Cases | 51 | 147 | 80 | 272 |  |  |
|  | HR (95% CI) | **1.24 (1.06 1.46)** | **0.87 (0.77 0.98)** | 1.11 (0.97 1.27) | 0.98 (0.91 1.06) | p=0.51 |  |
| Bladder | Cases | 41 | 145 | 45 | 262 |  |  |
|  | HR (95% CI) | 0.92 (0.74 1.15) | **1.12 (1.02 1.23)** | 0.94 (0.77 1.14) | 1.04 (0.97 1.12) | p=0.69 |  |
| Colorectal | Cases | 390 | 570 | 373 | 1040 |  |  |
|  | HR (95% CI)^e, g, f (males)^ | 0.99 (0.92 1.07) | 1.05 (0.99 1.10) | 1.06 (0.99 1.13) | 1.01 (0.98 1.05) | p=0.64 (females); p=0.64 (males) |  |
| Colon | Cases | 273 | 325 | 262 | 652 |  |  |
|  | HR (95% CI)^e, g, f (males)^ | 1.001 (0.92 1.09) | 1.03 (0.96 1.11) | **1.08 (1.002 1.17)** | 1.03 (0.98 1.07) | p=0.95 (females); p=0.72 (males) |  |
| Rectum | Cases | 97 | 232 | 100 | 378 |  |  |
|  | HR (95% CI)^e, g^ | 1.04 (0.89 1.20) | 1.04 (0.96 1.13) | 0.98 (0.86 1.13) | 1.003 (0.94 1.07) | p=0.45 (females); p=0.78 (males) |  |
| Brain tumours | Cases | 47 | 103 | 52 | 139 |  |  |
|  | HR (95% CI) | 0.90 (0.72 1.13) | 0.99 (0.86 1.13) | 1.11 (0.93 1.33) | 1.06 (0.96 1.16) | p=0.67 |  |
| Thyroid | Cases | 46 | 32 | 51 | 50 |  |  |
|  | HR (95% CI) | 1.09 (0.89 1.34) | 1.05 (0.83 1.32) | 0.97 (0.80 1.19) | 1.05 (0.89 1.24) | p=0.98 |  |
| Haematological malignancies | Cases | 293 | 513 | 269 | 698 |  |  |
|  | HR (95% CI) | 1.01 (0.93 1.10) | 1.04 (0.98 1.10) | 0.93 (0.86 1.01) | 1.01 (0.97 1.06) | **p=0.0004** |  |
| Non-Hodgkin’s lymphoma | Cases | 153 | 237 | 138 | 327 |  |  |
|  | HR (95% CI) | 1.05 (0.93 1.17) | 1.06 (0.98 1.15) | 0.94 (0.84 1.05) | 1.02 (0.96 1.09) | p=0.08 |  |

BFP: Body fat percentage; PA: Physical activity.

All models were adjusted for age, sex, ethnicity (white/other), deprivation index (quintiles), education (University degree, A-levels/HNC/HND/NVQ, GCSE/O-level/CSE, OTHER, None), fruit and vegetable intake (<5 portions/day, ≥5 portions/day), BMI (kg/m2), height (m), smoking status (never, former light smoker [<20 pack-years], former heavy smoker [≥20 pack-years], current light smoker [<20 pack-years], current heavy smoker [≥20 pack-years]) and alcohol intake (never, former, current [<once/week], current [≥once/week]).

^a^Additional site-specific covariates in the final model include use of sun/UV protection (Never/rarely/sometimes; most of the time/always; do not go out in sunshine).

^b^Additional site-specific covariates in the final model include HRT use (ever used/never used), oral contraceptive use (ever used/never used), number of live births (0, 1, 2, 3+ live births), age at menarche (early menarche [<12 years], menarche at 12-14 years, late menarche [≥15 years]), age at menopause (<40 years, 40-44 years, 45-49 years, 50-54 years, 55-59 years, 60-64 years, ≥65 years, not had menopause/unsure), hysterectomy status (had hysterectomy, not had hysterectomy/unsure).

^c^Additional site-specific covariates in the final model include HRT use (ever used/never used), oral contraceptive use (ever used/never used), number of live births (0, 1, 2, 3+ live births), age at menarche (early menarche [<12 years], menarche at 12-14 years, late menarche [≥15 years]), age at menopause (<40 years, 40-44 years, 45-49 years, 50-54 years, 55-59 years, 60-64 years, ≥65 years, not had menopause/unsure), hysterectomy status (had hysterectomy, not had hysterectomy/unsure).

^d^Additional site-specific covariates in the final model include diabetes at baseline (yes/no).

^e^Additional site-specific covariates in the final model include diabetes at baseline (yes/no), aspirin use (regular use/non-regular use or no use), HRT use (ever used/never used; females only), red meat intake (portion/week), processed meat intake (portion/week).

^f^Final model also adjusted for waist-hip ratio (>94cm in men, >80cm in women).

^f(males)^For cancer sites which were adjusted for different sets of covariates for males and females (colorectal, colon, rectum), this indicates that the final model for male participants was also adjusted for waist-hip ratio (>94cm in men).

^g^Results for males and females combined using meta-analysis as covariates are different.

^h^Final model also adjusted for family history of cancer (mother/father/sibling had cancer, no family history).

**Table 1.9. Results of Cox proportional hazards analyses investigating the association between a -hour increase in self-report daily TV viewing time and female-specific cancer incidence for subgroups defined by participant menopausal status**

|  |  | Not had menopause | Had menopause | Unsure | Wald test for interaction |  |
| --- | --- | --- | --- | --- | --- | --- |
| Breast (female only) | Cases | 1107 | 3104 | 246 |  |  |
|  | HR (95% CI)^a, c^ | 1.01 (0.97 1.06) | 1.003 (0.98 1.03) | 1.01 (0.92 1.10) | p=0.93 |  |
| Uterus | Cases | 112 | 604 | 32 |  |  |
|  | HR (95% CI)^b^ | 0.89 (0.77 1.02) | 0.99 (0.94 1.04) | 0.87 (0.66 1.13) | p=0.07 |  |
| Ovary | Cases | 81 | 395 | 70 |  |  |
|  | HR (95% CI) | 1.09 (0.94 1.26) | 1.02 (0.96 1.09) | 0.92 (0.79 1.08) | p=0.02 |  |

All models were adjusted for age, sex, ethnicity (white/other), deprivation index (quintiles), education (University degree, A-levels/HNC/HND/NVQ, GCSE/O-level/CSE, OTHER, None), fruit and vegetable intake (<5 portions/day, ≥5 portions/day), BMI (kg/m2), height (m), smoking status (never, former light smoker [<20 pack-years], former heavy smoker [≥20 pack-years], current light smoker [<20 pack-years], current heavy smoker [≥20 pack-years]) and alcohol intake (never, former, current [<once/week], current [≥once/week]).

^a^Additional site-specific covariates in the final model include HRT use (ever used/never used), oral contraceptive use (ever used/never used), number of live births (0, 1, 2, 3+ live births), age at menarche (early menarche [<12 years], menarche at 12-14 years, late menarche [≥15 years]), age at menopause (45-55 years, <40 years, 40-45 years, ≥55 years, not had menopause/unsure), hysterectomy status (had hysterectomy, not had hysterectomy/unsure).

^b^Additional site-specific covariates in the final model include HRT use (ever used/never used), oral contraceptive use (ever used/never used), number of live births (0, 1, 2, 3+ live births), age at menarche (early menarche [<12 years], menarche at 12-14 years, late menarche [≥15 years]), age at menopause (45-55 years, <40 years, 40-45 years, ≥55 years, not had menopause/unsure), hysterectomy status (had hysterectomy, not had hysterectomy/unsure).

^c^Final model also adjusted for family history of cancer (mother/father/sibling had cancer, no family history).

**Supplementary file 2: Testing linearity assumptions using restricted cubic splines**

Restricted cubic splines were used to facilitate detailed description of the dose-response curves for the following continuous exposure variables: daily TV viewing time, daily recreational computer time, daily total recreational screen time, daily moderate-intensity physical activity, daily vigorous-intensity physical activity, and daily walking time (1,2). Splines used five knots specified at the locations recommended by Harrell (2015; at the 5^th^, 27.5^th^, 50^th^, 72.5^th^, and 95^th^ percentiles) for daily TV viewing time, daily total recreational screen time, daily moderate-intensity physical activity, and daily walking time (3). Due to small sample sizes, four knots were specified for daily recreational computer time (at the 5th, 35th, 65th, and 95^th^ percentiles) and three knots were specified for daily vigorous-intensity physical activity (at the 10th, 50th, and 90^th^ percentiles) (3). Non-linearity of the dose-response curves was examined using likelihood ratio tests (p<0.05 provided evidence for potential violation of linearity assumptions). When likelihood ratio tests indicated potential violation of the linearity assumption, we examined for a non-linear association visually. Most likelihood ratio tests for the examined associations were non-significant, and visual inspection of spline graphs revealed no serious violation of linearity assumptions, although the relationship between daily total screen time and oesophagus cancer risk appears to be curvilinear. Therefore, we proceeded with our continuous variable analyses as planned.

1. Harrell Jr FE, Lee KL, Pollock BG. Regression models in clinical studies: determining relationships between predictors and response. J Natl Cancer Inst. 1988;80(15):1198–202.

2. Desquilbet L, Mariotti F. Dose-response analyses using restricted cubic spline functions in public health research. Stat Med. 2010 Apr 30;29(9):1037–57.

3. Harrell Jr FE. Regression Modeling Strategies With Applications to Linear Models, Logistic and Ordinal Regression, and Survival Analysis. Second Edi. Cham: Springer; 2015.

**Spline graphs and tests for non-linear associations**

Spline graphs and likelihood ratio (LR) tests for non-linear associations are presented below for associations where the likelihood ratio test indicated potential violation of the linearity assumption (p<0.05). Graphs show non-linear associations between the relevant exposures and cancer risk in the final adjusted models.

**Lung cancer, daily recreational computer time**

Reference value=0.5. LR test: Chi2(2)=7.89, p=0.0194.

**Lung cancer, daily moderate-intensity physical activity**

Reference value=0. LR test: Chi2(3)=15.56, p=0.0014.

**Lung cancer, daily vigorous-intensity physical activity**

Reference value=0. LR test: Chi2(1)=6.32, p=0.0120.

**Breast cancer, daily moderate-intensity physical activity**

Reference value=0. LR test: Chi2(3)=9.60, p=0.0223.

**Breast cancer, daily vigorous-intensity physical activity**

Reference value=0. LR test: Chi2(1)=7.97, p=0.0048.

**Prostate cancer, daily moderate-intensity physical activity**

Reference value=0. LR test: Chi2(3)=9.23, p=0.0263

**Oesophagus cancer, daily total recreational screen time**

Reference value=2.5. LR test: Chi2(3)=7.97, p=0.0466.

**Oesophagus and stomach cancer, daily moderate-intensity physical activity**

Reference value=0. LR test: Chi2(3)=9.72, p=0.0211.

**Colon cancer (males), daily vigorous-intensity physical activity**

Reference value=0. LR test: Chi2(1)=5.50, p=0.0190.

**Rectum cancer (males), daily moderate-intensity physical activity**

Reference value=0. LR test: Chi2(3)=8.38, p=0.0388.

**Hepatobiliary tract cancer, daily vigorous-intensity physical activity**

Reference value=0. LR test: Chi2(1)=9.81, p=0.0017.

**Bladder cancer, daily vigorous-intensity physical activity**

Reference value=0. LR test: Chi2(1)=7.58, p=0.0059.
